# Lexical Surprisal Shapes the Time Course of Syntactic Structure Building

**DOI:** 10.1162/nol_a_00155

**Published:** 2024-10-11

**Authors:** Sophie Slaats, Antje S. Meyer, Andrea E. Martin

**Affiliations:** Max Planck Institute for Psycholinguistics, Nijmegen, Netherlands; Department of Basic Neurosciences, University of Geneva, Geneva, Switzerland

**Keywords:** delta, entropy, MEG, surprisal, syntax, temporal response functions

## Abstract

When we understand language, we recognize words and combine them into sentences. In this article, we explore the hypothesis that listeners use probabilistic information about words to build syntactic structure. Recent work has shown that lexical probability and syntactic structure both modulate the delta-band (<4 Hz) neural signal. Here, we investigated whether the neural encoding of syntactic structure changes as a function of the distributional properties of a word. To this end, we analyzed MEG data of 24 native speakers of Dutch who listened to three fairytales with a total duration of 49 min. Using temporal response functions and a cumulative model-comparison approach, we evaluated the contributions of syntactic and distributional features to the variance in the delta-band neural signal. This revealed that lexical surprisal values (a distributional feature), as well as bottom-up node counts (a syntactic feature) positively contributed to the model of the delta-band neural signal. Subsequently, we compared responses to the syntactic feature between words with high- and low-surprisal values. This revealed a delay in the response to the syntactic feature as a consequence of the surprisal value of the word: high-surprisal values were associated with a delayed response to the syntactic feature by 150–190 ms. The delay was not affected by word duration, and did not have a lexical origin. These findings suggest that the brain uses probabilistic information to infer syntactic structure, and highlight an importance for the role of time in this process.

## INTRODUCTION

To understand language, we must recognize words and combine them into larger linguistic units like phrases and sentences. This process is complicated by the fact that as the sensory input unfolds, be it speech, sign, or text, we must settle on an interpretation of the input (viz., perception and recognition) in addition to transforming or combining that input into larger meaning units. At least two types of information can help us in this process, knowledge about what we are perceiving (e.g., which linguistic unit, how that unit fits with others) and knowledge about how likely it is to occur. These two types of information can be roughly described as the structure of language and knowledge of its statistical distribution. Over the past several decades, much psycholinguistic research has focused on accounting for syntactic phenomena either as a form of transitional probabilities between different linguistic units (Frank & Bod, 2011; Frank & Christiansen, 2018; Frost et al., 2019; McCauley & Christiansen, 2019), or as a separate level of representation that is hierarchically structured and abstracts away from the lexical items itself (e.g., Brennan & Hale, 2019; Lo et al., 2022; Matchin & Hickok, 2020), without much integration between the two types of knowledge. Nevertheless, recent work in psycho- and neurolinguistics has provided evidence that both types matter (Maheu et al., 2022; Nelson, El Karoui, et al., 2017; Weissbart & Martin, 2024). We know from perception and cognition that brains, both human and non-human, are probabilistic engines (e.g., Santolin & Saffran, 2018) that are capable of producing abstract, generalizable representations (e.g., Coopmans et al., 2023; Deacon, 1997; Doumas et al., 2008; Martin & Doumas, 2019). Here, therefore, we test a framework where humans use lexical distributional information to build abstract, hierarchical representations that give rise to meaning. It is an instantiation of cue integration (viz., Ernst & Bülthoff, 2004; Marslen-Wilson & Tyler, 2007; Martin, 2016, 2020); word-by-word statistics are cues for linguistic rules.

### Statistical Patterns in Learning and Comprehension

Psycholinguistic experiments have shown that humans are capable of rapidly extracting statistical regularities; infants and adults alike are able to extract words, simple rules, and even nonadjacent dependencies from a continuous stream of syllables after as little as 2 min of exposure using nothing more than transitional probabilities (Aslin et al., 1998; Batterink & Paller, 2017, 2019; Gervain, 2014; Gómez, 2002; Isbilen et al., 2022; Saffran et al., 1996; Vouloumanos & Werker, 2009). It has since been hypothesized that this capacity underlies our extraction of syntactic rules; we use distributional cues to infer the structure underlying the input (Rowland et al., 2012; Saffran, 2001; Thompson & Newport, 2007). Early modeling work revealed exactly these statistical patterns that language follows are a direct consequence of the syntactic structure of the input (e.g., Elman, 1991, 1993). More recently, corpus studies and computational models suggest that (backward) surprisal contains information about the phrase structure of sentences (McCauley & Christiansen, 2019) and that large neural networks can capture statistical patterns associated with hierarchical structure (Kuncoro et al., 2018; Lakretz et al., 2019; Manning et al., 2020).

These findings culminated in models using those statistical patterns not just in a theory of language acquisition, but also in a theory of language comprehension. An influential example of such a theory is surprisal theory (Hale, 2001, 2006, 2016; Levy, 2008a, 2008b; Levy & Gibson, 2013). Surprisal theory broadly aims to predict when comprehension difficulties arise. The underlying assumption is that comprehenders make use of probabilistic information to predict both the structure of the input they have just heard or seen and what they might encounter next. The extent to which these predictions are correct is hypothesized to determine the difficulty of processing. The model uses surprisal, the negative log probability of a word (or other linguistic unit) given the context, as a quantification of the validity of the predictions made. If surprisal is high on a given word, this word was unexpected given the context, and processing difficulty (often indexed by slower reaction times (RTs) in, for example, self-paced reading tasks) is predicted to occur. Since surprisal can be calculated over any representation, be it phonemic, lexical, or even structural, surprisal theory does not commit to a representation of language. It is agnostic about the representations and mechanisms that lead to structure-dependent interpretation (see Slaats & Martin, 2023).

Since the introduction of surprisal theory, distributional information has been shown to account for much variance in models of behavior and neural activity *after* learning as well (Armeni et al., 2019; Brennan & Hale, 2019; Hale, 2006, 2016; Hasson, 2017; Hasson & Tremblay, 2016; Heilbron et al., 2022; Levy & Gibson, 2013; Smith & Levy, 2013), a trend that continues rapidly with the introduction of large language models. For example, higher surprisal values and a larger decrease in entropy are both associated with slower reading times (Aurnhammer & Frank, 2019; Frank, 2013; Linzen & Jaeger, 2016)—the signature of processing difficulty from surprisal theory. More recently, neuroimaging experiments have shown that oscillations at delta, beta and gamma bands track surprisal (Weissbart et al., 2020), entropy reduction correlates with temporal lobe activity (Nelson, Dehaene, et al., 2017), and surprisal and word frequency are tracked over and above acoustic and speech segmentation representations (Gillis et al., 2021). In other words, probability at the word level is a good predictor for behavioral and neurophysiological measurements (Slaats et al., 2023).

Much work on surprisal and other distributional estimates aims to model processing difficulty and does not draw explicit conclusions on mental representations that underlie the observed effects. This is because probability can be calculated over any representation, and the resulting estimates are necessarily affected by latent linguistic constraints that guide the input, such as syntax (Slaats & Martin, 2023). This makes it difficult to decide whether the origin of the effects lies in the probabilistic relation between words, or in other representations that play a role in language.

We have seen above that probabilistic language models can statistically approximate aspects of syntactic structure (Kuncoro et al., 2018; Lakretz et al., 2019; Manning et al., 2020; Slaats & Martin, 2023). This opens up the possibility that some effects that are attributed to probabilistic processing may be due to structural processing instead. However, when we look at experimental data, we observe that the converse also holds; some effects that are attributed to linguistic structure can be evoked by statistical regularities as well. In a seminal work, Ding and colleagues (2016) showed that the occurrence rate of linguistic structures (syllables, phrases, and sentences) in speech are reflected in power in the neural signal at the corresponding frequencies (4 Hz, 2 Hz, and 1 Hz, respectively). This effect was widely adopted in the literature as reflecting the construction of linguistic units; the brain encodes abstract linguistic information. However, since its publication several studies have shown that the low-frequency tagging effects can be induced by transitional probability information alone (Bai, 2022; Batterink & Paller, 2017).

This overwhelming evidence for the importance of distributional information has reignited the debate on the abstract representations of language, namely whether language acquisition and language comprehension alike are both rooted in sequential, statistical information (Frank et al., 2012; Frank & Bod, 2011; Frank & Christiansen, 2018; Frank & Yang, 2018), rather than the hierarchical tree structures that are part of linguistic theory (Chomsky, 1956, 1965; Everaert et al., 2015; Pollock, 1989; Rizzi, 1997, i.a.). This is quite the departure from the early hypothesis in the statistical learning literature that statistics function as a cue rather than the instantiation of the structure itself.

### Syntactic Structure in Neural Dynamics

Logically, however, statistics-only accounts struggle to explain language behavior (Fodor & Pylyshyn, 1988; Hale et al., 2022; Martin, 2016, 2020; Slaats & Martin, 2023). For one, listeners are able to understand sentences that include words or combinations of words that they never encountered before. In line with this observation, there is ample evidence for the use of abstract structure in language learning and comprehension. For example, learners privilege abstract knowledge of scope-taking over transitional probabilities when presented with a structurally altered version of English (Culbertson & Adger, 2014) and are able to infer abstract structure in the input after a single exposure (Marcus et al., 1999). Beyond that, everyday language production shows that both children and adults produce utterances that they have not heard before (Conwell & Demuth, 2007; Valian, 1986).

And indeed, neuroimaging studies have shown repeatedly that inferred structural information modulates activity in low frequency bands, particularly the delta (<4 Hz) band (Bai et al., 2022; Brennan & Martin, 2020; Kaufeld et al., 2020; Lo et al., 2022; Meyer et al., 2017; Tavano et al., 2022; Ten Oever et al., 2022) and gamma band (Nelson, El Karoui, et al., 2017; Peña & Melloni, 2012)—even when there are no acoustic markers of this structural information. For example, Bai and colleagues (2022) presented participants with two different structures: phrases (*de blauwe bal*, the blue ball) and sentences (*de bal is blauw*, the ball is blue). These two types of stimuli had the same number of syllables and indistinguishable power spectra, but the neural response differed between the conditions in various ways: low-frequency (1–8 Hz) phase coherence, <2 Hz phase connectivity, and theta–beta (4–10 Hz, 15–40 Hz, respectively) phase-amplitude coupling. These findings suggest that even small changes of syntactic structure have large consequences for the (low-frequency) neural dynamics. Similarly, Tavano and colleagues (2022) show that those syntactic categories, phrases and sentences, generate a neural rhythm as reflected in inter-trial phase coherence that is mathematically independent of the presentation rate of the words.

Evidence for delta-band involvement in the process of structure building also comes from studies comparing word lists and sentences (Lo et al., 2022; Lu et al., 2022; Slaats et al., 2023). Lu and colleagues (2022) presented participants with sentences and word lists of animate and inanimate nouns that both repeated at 1 Hz to assess whether delta-band dynamics track semantic properties of words, or whether the changes are related to structural properties of the stimulus. A lexical distributional approach as those advocated by Frank and colleagues (Frank et al., 2012; Frank & Bod, 2011; Frank & Christiansen, 2018; Frank & Yang, 2018) would predict stronger 1 Hz and 2 Hz response peaks in the word list condition than in the sentence condition; the opposite is predicted by model that assumes a role for syntactic structure in delta-band activity. The study showed that the 1 Hz response peak was larger for sentences than for word lists, suggesting again that low-frequency activity is modulated by or causal for structure building. In a similar vein, Lo and colleagues (2022) showed that synchronization to the sentential rhythm in the delta band only occurs when the sentences are syntactically well formed. Finally, Slaats and colleagues (2023) compared delta-band responses to individual words between word lists and sentences, while controlling for effects of surprisal. This study showed that responses to words were affected in their temporal and spatial organization when embedded in a sentence structure; the responses appeared earlier and activity was propagated to left inferior frontal areas in the sentence condition only.

### A Time and Space for Both

Some studies pit the importance of structure or sequential probabilities against each other (Brennan & Hale, 2019; Christiansen & Chater, 2016; Frank et al., 2012; Frank & Yang, 2018). Frank and Bod (2011), for example, used probabilistic language models that were trained to predict the next part-of-speech (POS) with a hierarchical and sequential architecture to model reading time data. They found that the hierarchical models did not account for variance over and above sequential probability estimates, and suggested that human sentence processing relies more on sequential than on hierarchical structure. Brennan and Hale (2019), on the other hand, suggest that hierarchical structure is important during language comprehension. They use several sequential models and a context-free grammar to obtain surprisal for POS and use those to model electroencephalography (EEG) data from naturalistic listening. In contrast to Frank and Bod (2011), they find that the context-free grammar estimates predict EEG data over and above the sequential models.

When we consider all of these findings together, we must conclude that both distributional and abstract, hierarchical syntactic information play a role in language comprehension—and that both shape the neural signal. Indeed, a study by Roark et al. (2009) showed that models with separate lexical and syntactic surprisal/entropy features are better at modeling RT data than models that do not make this distinction. Similarly, Nelson, El Karoui, et al. (2017) found that intracranial EEG signals differentially encode responses to probabilistic and syntactic information. In line with these findings, several (statistical) learning experiments suggest that the brain represents the statistical biases as well as abstract rules (Maheu et al., 2022; Monte-Ordoño & Toro, 2017; Saffran, 2001; Toro et al., 2011). A model in which probability plays a role, while structure does too, is in line with one of the brain’s main features; it can map probabilistic information onto deterministic representations (Martin, 2020; Tenenbaum et al., 2011; a relatively undisputed example is categorical perception; Harnad, 2003).

### The Current Study

Given this background, rather than contrast distributional information with syntactic information, we investigate how these factors jointly shape the neural signal. As in the statistical learning literature (e.g., Saffran, 2001; Thompson & Newport, 2007), we ask if distributional information can serve as a cue for syntactic structure during comprehension; contextual lexical distributional information should affect the quality of the neural signature of structure building. Lexical probability thus should interact with abstract representations of sentence structure. We ask (1) whether the neural encoding of linguistic structure changes as a function of the distributional properties of a word, and (2) whether this influence can be linked to probabilities in the immediate context (two preceding words) or rather to probabilities in the larger context. Following findings from statistical learning and models of transitional probabilities (McCauley & Christiansen, 2019; Thompson & Newport, 2007) and in line with a model of language comprehension proposed by Martin (2016, 2020) we hypothesize that the neural encoding of linguistic structure is affected by the probability of a word in the context.

While this may appear a relatively straightforward question to answer, several issues of both methodological and theoretical origin arise. The first issue concerns the operationalization of contrasting a distributional factor with a latent structural one. Problems arise because of the nature of lexical distributional information such as surprisal and entropy: these values are affected by any change that is made in the underlying structure (Slaats & Martin, 2023). If one wants to manipulate the latent syntactic structure underlying a sentence, the surprisal values of the words will also change. Because of this issue, the potential effects at hand are not easily captured in a factorial design. We solve this problem by making use of variance of lexical distributional information and syntactic structures that occurs naturally in continuous speech; we use temporal response functions (TRFs) to model responses to latent linguistic variables, such as syntactic structure and lexical surprisal, in magnetoencephalography (MEG) data obtained using a naturalistic listening paradigm.

The second issue concerns the broader theoretical questions concerning the nature of distributional information in the brain: Over which representation does the brain store distributional information, and how is this implemented mechanistically? While this particular study will not provide an answer to this larger question, it will speak to two questions that follow from it. Specifically, (a) which type of distributional information most faithfully captures the information available to the brain, and (b) which of those plays a role in the process of the inference of latent linguistic structure? These questions concern estimates derived from large language models like the GPT family, those derived from simpler models like long-short term memory networks, or the even simpler trigram models. In this study we look into a version of GPT2 and a trigram model.

Separate predictions may be derived with regards to the two questions posed above. With respect to question (a), one can expect models with a larger context and potentially enhanced sensitivity to the latent factors driving the statistical patterns to perform better when it comes to describing the neural signal generally as a consequence of capturing more sources of variance—and indeed, this appears to be true (Heilbron et al., 2019) until a certain point (Kuribayashi et al., 2022; see also Futrell et al., 2020). A larger context and more parameters theoretically allow for capturing more fine-grained sources of variance. Predictions concerning question (b), on the other hand, are not so easy to derive. While we hypothesize generally that lexical distributional information can affect the process of syntactic structure building as described above, it is unknown whether one would need long-context, fine-grained variability to capture this hypothesized effect, or whether the local context provides enough distributional information to capture it. The statistical learning literature suggests that short-context probability, such as bigram and trigram frequencies, can function as a cue for linguistic structure (Aslin et al., 1998; Aslin & Newport, 2012; Frost et al., 2019; Gómez, 2002; Isbilen et al., 2022; Knowlton & Squire, 1996; McCauley & Christiansen, 2019; Thompson & Newport, 2007). For this reason, we hypothesize that any effect of lexical distributional information on the inference of syntactic should be observable using a short-context metric such as trigram probability.

In summary, in the present study, we address the following questions: (1) whether the neural encoding of linguistic structure changes as a function of the distributional properties of a word, and (2) whether this influence can be linked to probabilities in the immediate context (two preceding words) or rather to probabilities in the larger context. We do this by analyzing MEG data of participants who listened to fairytales. Using TRFs, we model the neural signatures of syntactic structure building in the delta band, and compare those between different distributional contexts (i.e., high versus low surprisal). In order to characterize the lexical distributional information that is available to the brain, we estimate lexical distributional information with two different language models: a trigram model, which uses only two words to estimate the predictability of the current word, and a Dutch version of GPT2, a large transformers model that uses a very large context window.

## MATERIALS AND METHODS

### Participants

24 right-handed native speakers of Dutch (18 female, 20–58 years old; mean = 33.4) were recruited from the participant pool at Radboud University Nijmegen, the Netherlands. All participants reported normal hearing, had normal or corrected-to-normal vision, and reported no history of language-related impairments. Participants gave written informed consent. The experiment was approved by the Ethics Committee for human research Arnhem/Nijmegen (project number CMO2014/288).

### Materials

The stimuli consisted of three fairytales (one by Hans Christian Andersen, two by the Brothers Grimm) read out at comfortable pace by female native speakers of Dutch. Each story was divided into segments of approximately 5.5 min (range 4 min 58 s–6 min 40 s), leading to nine segments and a total duration of 49 min and 17 s. Each segment was normalized for loudness using the ffmpeg software (EBU R128 standard; FFmpeg Developers, 2024). The transcripts of the stories were checked for consistency with the recordings, adjusted for spelling where necessary and subsequently automatically aligned with the audio using the WebMAUS segmentation software to extract word onset time-points (Kisler et al., 2017). All stories contained a natural variation of words and sentence structures. In total, the stories contained a total of 8,551 words in 791 sentences, with an average length of 10.8 words (range 1–35, *SD* 5.95).

### Procedure and Data Acquisition

Participants were tested individually in a magnetically shielded room. They were instructed to sit still and look at a fixation cross that was presented in the middle of a screen while they listened passively to the fairytales. Each block started with a 10 s period during which resting state data were recorded. After each story segment, five multiple-choice comprehension questions were asked. On average, participants’ accuracy was 88.1% (*SD* 7.52%), indicating that they were paying attention to the content of the stories. The stimuli were presented via plastic tubes and ear pieces to both ears. The experiment was run using Psychtoolbox in Matlab (Brainard, 1997).

MEG data were recorded continuously with a 275-channel axial gradiometer system at a sampling frequency of 1200 Hz. Three head localizer coils were attached to the participant’s head (nasion and left and right ear canals through fitted ear molds) to determine the position of the head relative to the MEG sensors. The head position was monitored throughout measurement and, if necessary, corrected during breaks. In addition, eye movements and heartbeat were recorded with additional electrooculography (EOG) and electrocardiography (ECG) electrodes.

### MEG Preprocessing

Preprocessing was done with MNE-Python (Version 0.23.1; Gramfort et al., 2013). The MEG data were down sampled to 600 Hz and band-pass filtered at 0.5–40 Hz using a one-pass zero-phase, noncausal finite impulse response filter. We interpolated bad channels using Maxwell filtering, and used independent component analysis to eliminate artifacts resulting from eye movements (EOG) and heartbeats (ECG). The data were segmented into nine epochs time-locked to the onset and offset of the story audio recordings. At some point in data collection, some channels of the scanner failed due to technical issues. We interpolated these channels for those participants to ensure the same number of channels for all participants. In continuation, the epochs were resampled to 200 Hz and band-pass filtered between 0.5 Hz and 4 Hz (the delta band).

### Temporal Response Functions

We modeled the neural signal using TRFs with different acoustic and linguistic features. This approach has been used to distinguish between responses to different linguistic features, ranging from the speech envelope and phonemic information (Di Liberto et al., 2015; Donhauser & Baillet, 2020; Tezcan et al., 2023) to lexical information (Slaats et al., 2023; Weissbart et al., 2020) and even to syntactic embedding (Nelson, El Karoui, et al., 2017). In essence, the method is a multivariate multiple linear regression, where we used lagged time series of different annotations of the stimulus as features. In this way, it is possible to distinguish between variability in the signal that stems from acoustic processing, lexical processing, and many others.

The equation of the model reads as follows:

$$y_{c}\left(t\right)=\sum \sum x_{f}\left(t\right)\beta_{f}\left(t-\tau_{k}\right)+\eta \left(t\right)$$ (1)

where {*y*_*c*_}_*t*_, {*x*_*f*_}_*t*_, {*β*_*f*_}_*t*_ represent the recorded MEG signal of a given channel c, the input feature f and its TRF, respectively. {*η*}_*t*_ is a Gaussian noise process which accounts for aspects of the stimulus that are not captured by the coefficients attributed to the features in the model. We solved this equation using ridge regression (as opposed to, for example, boosting; Brodbeck et al., 2023). This means that we estimated the coefficients of the TRFs $\hat{\beta_{f}}$ by minimizing the squared error between the measured MEG signals and the reconstructed signal obtained from Equation 1 while keeping the norm of the TRFs’ coefficients ∥*β*∥_2_ low to avoid overfitting. This minimization problem is solved in a closed form by:

$$\hat{\beta }={\left(X^{T}X+\lambda I_{d}\right)}^{-1}X^{T}Y$$ (2)

where *Y* ∈ ℝ^*N*×*C*^ is the matrix representation of the measured MEG signal (for *C* channels arranged column-wise, each with *N* data samples); $\hat{\beta }$ ∈ ℝ^(*K*.*F*)×*C*^ contains the estimated TRFs with *K* lags, *F* features for all *C* channels; *X* ∈ ℝ^*N*×(*K*.*F*)^ is a matrix containing all lagged feature time series of length *N*; *λ* is a regularization coefficient and *I*_*d*_ the identity matrix. The regularization coefficient is needed to avoid overfitting, which in this case translates to the square matrix *X*^*T*^*X* not being full rank. Numerically small eigenvalues or simply ill-conditioned matrices can make the inversion unstable and therefore require regularization. In TRF models, this happens when features present some amount of autocorrelation, as is the case in our models (e.g., the acoustic envelope is strongly autocorrelated).

In Equation 1, the vector of weights *β*_*f*_(*t*) represents the coefficients parameterizing the TRFs. They form a time course reminiscent of an event related potential that tells us at which point in time (and, potentially, where) a feature modulates the neural signal. Thus, an increase at a certain lag for a given feature reflects an increase in the associated brain response to this feature at that given sensor and at the given time lag after stimulus onset.

To evaluate how our models perform at reconstructing the neural data, we computed the Pearson’s correlation coefficient between the true data and data reconstructed using the estimated TRFs. The correlation between the reconstruction and the original MEG indicates how much of the variance in the neural signal is explained by the features. The TRFs were not estimated on the same portion of data used to score the model. As further explained in Model Fitting and Statistical Analysis, we used a nested cross-validation procedure to tune the regularization parameter, estimate the TRF coefficients, and finally score the resulting model. Unless specified otherwise, all analyses described below were done with custom made Python scripts using MNE-Python (Gramfort et al., 2013).

### Stimulus Representations

To characterize the speech signal and latent linguistic features, we constructed eight features that belong either to the base features or to the set of experimental features. The base features are present in every model, and are used to remove variance from factors that could potentially influence the results.

#### Base features

The base features are speech envelope, word onset, and word frequency.

The *speech envelope feature* was computed for each stimulus by taking the absolute value of the Hilbert transform and down sampling it to 200 Hz to match the MEG sampling rate. The envelope feature was added to represent the acoustic response and as such separate acoustic processing from linguistic processes of interest: structure building.

The *word onset feature* was added to capture broadly any time-locked response to word onset for which the variance is not already explained by other features. To this end, we extracted word onset time-points using the WebMAUS segmentation software (Kisler et al., 2017). We used a train of unit impulses, where the feature signal is one at the word onset sample and zero otherwise:

$$x\left(t\right)=\sum_{\text{words}}\delta \left(t-t_{\text{onset}}\right)$$ (3)

These impulse trains were convolved with a Gaussian kernel with a standard deviation of 15 ms. Such temporal smoothing has the effect of inflating the autocorrelation of the signal. We designed the width of this smoothing such that the smoothed impulses end up with energy spanning a comparable frequency band as to our continuous regressor (the speech envelope). The Fourier transform of a Gaussian is also a Gaussian, and the 15 ms standard deviation of the temporal smoothing kernel equates to a spectral standard deviation of 21.22 Hz. This ensured that all features required a similar degree of regularization in the regression analysis and made it possible to include impulse-like features such as word onsets and the envelope in the same regularized regression. Notably, this also translates into some uncertainty about or knowledge of the exact word onset timings.

Like the word onset feature, the *word frequency feature* was constructed as an impulse train of zeros everywhere but at word onset. Here we used the respective word frequency value to modulate the height of the impulses. We used the log-transformed value of occurrence per million words, obtained from the SUBTLEX-NL corpus (Keuleers et al., 2010):

$$x_{wf}\left(t\right)=\sum_{\text{words}}-\log\left(P\left(w\right)\right)\times \delta \left(t-t_{\text{onset}}\right)$$ (4)

where *P*(*w*) represents the unigram probability estimated from occurrence per million words. If a word did not exist in the corpus, the fallback value of 0.301 (log/million) was used, corresponding to the lowest word frequency in the corpus. The values were scaled (divided by their standard deviation) across all stimuli. The resulting signal was convolved with the same Gaussian kernel as the word onset feature.

#### Experimental features

We designed five experimental features to investigate the influence of contextual lexical distributional measures on structure building: surprisal and entropy, the distributional features; and top-down, bottom-up, and left-corner node counts, the structural features.

The *surprisal feature* reflects how predictable a given word is in its context. It is the (traditionally two-based) log-transformation of the conditional probability of a word. If surprisal is low, the word was predictable given the context; if it is high, the word was not predictable given the context.

$$I\left(w\right)=-\log_{2}\left(P\left(w_{i}|w_{i-1}\ldots w_{i-n}\right)\right)$$ (5)

The *entropy feature* consists of lexical entropy, a weighted probability measure that quantifies the uncertainty about the upcoming word on the basis of the previous words. It provides a numeric answer to the following question: Given the *n* previous words, with what degree of certainty can we predict the upcoming word?

$$H\left(w_{i}\right)=-\sum_{k}P\left(w_{k}|w_{i-1}\ldots w_{i-n}\right)\times \log_{2}\left(P\left(w_{k}|w_{i-1}\ldots w_{i-n}\right)\right)$$ (6)

We generated these two metrics in two ways. For the long-context distributional information models, the values were derived from GPT2, a large-scale transformers language model that was fine-tuned for Dutch (de Vries & Nissim, 2021). This version of GPT2 was fine-tuned using a context window of 128 tokens. Tokens do not map onto words in a one-to-one fashion. Instead, a token can correspond to a word, but also to a morphological marker; for example, the Dutch plural “s” marker may be a token. As such, the context used for the surprisal and entropy estimates will roughly correspond to a little under 128 words. For the short-context distributional information models, the values were obtained from a trigram model created with SRILM (Stolcke, 2002) trained on ∼1.2 million words from the Dutch corpus from OpenSubtitles (Lison & Tiedemann, 2016). This model takes the preceding two words to estimate the surprisal (and entropy) values of the target word. We used Kneser-Ney discounting with interpolation to estimate values for missing words or trigrams.

**Figure 1.**
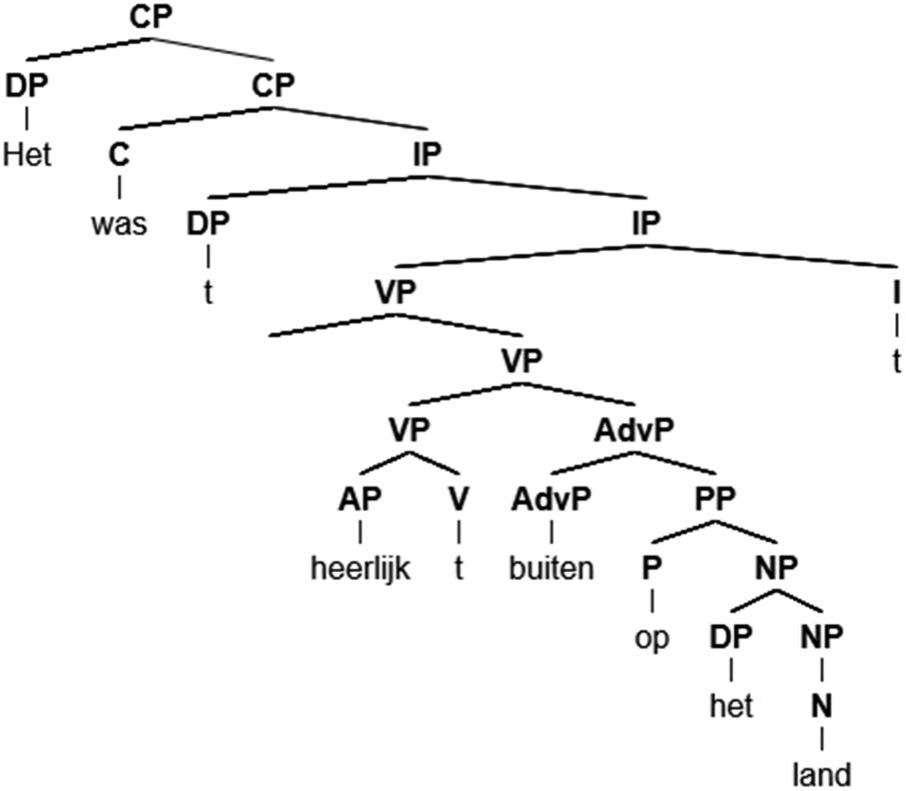
A parsed sentence from the stimuli. The sentences were parsed according to an adapted minimalist paradigm. The sentence reads (nonliterally): “The weather was lovely on the countryside.” This is the first sentence of one of the stories by Anderson. *Abbreviations*: CP = complementizer phrase; DP = determiner phrase; C = complementizer; IP = inflectional phrase; VP = verb phrase; I = inflection; AdvP = adverb phrase; AP = adjective phrase; V = verb; PP = prepositional phrase; P = preposition; NP = noun phrase; N = noun.

To extract neural signatures of structure building, we needed a feature that reflected the syntactic structure underlying the input. To this end, we manually parsed all sentences using a simplified version of X-bar theory (Carnie, 2013). This entailed that we created a full X-bar structure for all noun phrases (NPs) and verb phrases (VPs), but not for the other phrases unless intermediate projections were filled. Using the full X-bar structure for NPs and VPs ensured that each parse contained an explicit distinction between arguments and adjuncts, with arguments being attached as a sister of the head and adjuncts occupying the intermediate projection. An example of one of the resulting parses is displayed in Figure 1.

**Figure 2.**
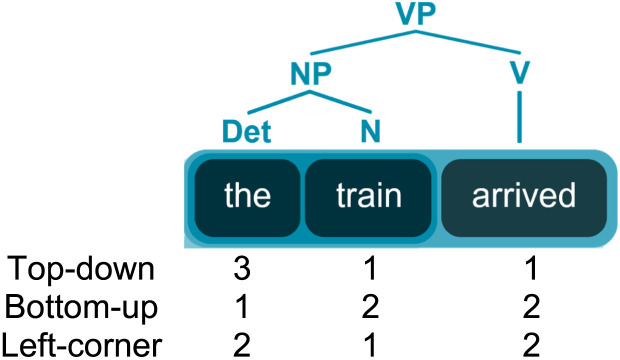
Node counts per word according to top-down, bottom-up, and left-corner parsing algorithms.

From these parses we extracted node count estimates to function as the syntactic features in our TRF models. Node counts have been found to effectively represent syntactic complexity in the neural signal (Brennan et al., 2016; Giglio et al., 2024; Li & Hale, 2019; Nelson, El Karoui, et al., 2017). Node counts can be computed in different ways, depending on the algorithm the parser is hypothesized to use to reach the structured representation. We calculated node counts according to three algorithms: a top-down algorithm (further: top-down), a bottom-up algorithm (further: bottom-up) and a left-corner algorithm (further: left-corner). The *top-down* parsing method is maximally predictive. Upon encountering a word, all nodes governing this word to the right are assumed to be built. For example, if the parser encounters the determiner “the” in the sentence “the train arrived” (see tree structure in Figure 2), the parser will build not only the determiner, but also the NP and the VP, hence a node-count of three. The *bottom-up* method is completely nonpredictive: in this method, the parser will build only the nodes it has seen all evidence for. That means that the NP from our example will not be built until the noun “train” has been seen. The *left-corner* algorithm is a mixture of these two. This mildly predictive parsing method will project a constituent as soon as the first item is found, but no constituents above this are built. In the case of the train, this means that the NP is built when the determiner has been seen, but the VP will only be built once the whole NP has been seen.

As can be seen in Figure 1, the parses we created contained traces of moved elements. These traces do not have acoustic correlates in the signal. In order to represent the structure they are part of, we assigned their node counts to other words within the sentence. Specifically, we added the node count of the trace to the node count of the word following it. This strategy was chosen because we reasoned that the location of these traces can be inferred after their position.

Since the linguistic features (frequency, entropy, surprisal, bottom-up node count, top-down node count, left-corner node count) might be correlated to some extent, we need to assert that the degree of multicollinearity present in our stimulus representation will not hinder the TRF coefficient interpretation. We checked whether the variance inflation factor (VIF) was below 5 (considered a relatively conservative measure of multicollinearity; Sheather, 2009; Tomaschek et al., 2018). The VIF was computed by correlating the z-scored entropy, surprisal, word frequency, and node count values and taking the diagonal of the inverted correlation matrix. This was done for all the stimuli. The VIF was higher than 9.7 for left-corner due to high positive correlations with both bottom-up and top-down. We did not include left-corner in our models. After removal of this feature, all VIF values were lower than 3.5 (see Supplementary Materials A in the Supporting Information, available at https://doi.org/10.1162/nol_a_00155, for the correlation matrices of the used features). Like the word frequency feature, the other features were scaled and inserted in a stick function, after which the stick function was convolved with the same Gaussian window.

### Model Fitting and Statistical Analysis

Any TRF analysis has two deliverables: first, the TRF (the development of the estimated coefficients across time), which is an ERP-like waveform that captures how the neural signal changes as a function of a feature of interest (in our case, the features of interest are the node-count responses); and, second, the reconstruction accuracy. This is a metric of model fit (Pearson’s correlation, as explained in Temporal Response Functions, above). We use the second deliverable, the reconstruction accuracy, to assess whether our used features are relevant for a description of the neural signal, and the first deliverable to evaluate whether syntactic structure building processes are affected by the lexical distributional context.

The current analysis consists of two parts: the main effects analyses, and the interaction analyses. In the main effects-part, we conducted an analysis on the reconstruction accuracy across the whole scalp to assess the contribution of each of the features individually, as well as a comparison between the effects of surprisal and entropy from our different language models (trigram and GPT2). We did this to ensure all the effects that were included in the interaction analysis were relevant for the neural signal, that is, to ensure that main effects were present before we investigated interactions. In the interaction-part, we conducted analyses on the TRFs from models that consider the interaction between estimates of lexical probability and syntactic structure building. The two parts are described in more detail below.

In the main effects analyses, we estimated TRF models for all combinations of the features of interest over and above a null model that included the envelope, word onset and word frequency features. The models and their features are summarized in Table 1. See Supplementary Materials A, for the correlation matrices of the lexical features. All models were fitted with surprisal and entropy features estimated from a trigram model and GPT2.

**Table 1.** The fitted encoding models in the main effects analyses

Model name	Feature						
	Envelope	Word onset	Word frequency	Surprisal	Entropy	Bottom-up	Top-down
main_null	X	X	X				
main_surprisal	X	X	X	X			
main_entropy	X	X	X		X		
main_distributional	X	X	X	X	X		
main_topdown	X	X	X				X
main_bottomup	X	X	X			X	
main_topdown_bottomup	X	X	X			X	X
main_surprisal_topdown	X	X	X	X			X
main_surprisal_bottomup	X	X	X	X		X	
main_surprisal_topdown_bottomup	X	X	X	X		X	X
main_entropy_topdown	X	X	X		X		X
main_entropy_bottomup	X	X	X		X	X	
main_entropy_topdown_bottomup	X	X	X		X	X	X
main_distributional_topdown	X	X	X	X	X		X
main_distributional_bottomup	X	X	X	X	X	X	
main_distributional_topdown_bottomup	X	X	X	X	X	X	X

*Note*. X indicates that a feature was included in the model.

Estimating all feature combinations allowed us to estimate a slope of the given feature irrespective of the presence of other features. We did this by averaging the reconstruction accuracies of the resulting model across sensors (i.e., one value per participant per model) and submitting these averages to linear mixed models using the lme4 in R (Bates et al., 2015). These models contained a binomial factor for each of the features of interest (surprisal, entropy, bottom-up, and top-down), indicating whether or not a feature was present in the model. We estimated large linear mixed effects models in which all factors interacted with each other. A model with a full random effects structure was not possible (because there were not enough observations), so we fit this large model four times with each time three out of four factors in the random effects structure. On each large model we performed model comparison using the *step* function from the LmerTest package (Kuznetsova et al., 2017). This function reduces the random- and fixed-effects structure of a model in a maximal-to-minimal fashion. We then compared the resulting best models for their Akaike information criterion (AIC) value, and report the model with the lowest AIC value below.

Because the effects of every feature may differ across the scalp, we also estimated the slope of every feature by averaging the per sensor reconstruction accuracy values over all the models that did or did not include a given feature. For example, to examine the effect of entropy, we averaged per participant, per sensor over all the models that include entropy to obtain one with-entropy value for every sensor for every participant, and we averaged per participant, per sensor over all the models that do not include entropy to obtain one without-entropy value for every sensor for every participant (see Table 1). Per feature, that means we obtained two values for every sensor: one with the feature, and one without. We then evaluated any difference between these using a cluster-based permutation test between these values using permutation_cluster_test from the MNE-Python library.

Cluster-based permutation tests address the null hypothesis of exchangeability across conditions by a Monte Carlo estimate of the randomization distribution of a cluster-based test statistic, optimizing statistical sensitivity while controlling the false alarm rate. Here, we used the *t* statistic as the test statistic. In these tests, we create matrices of all sensors and (in the case of TRF waveforms) samples. Then, we compute the difference between two conditions and express it as a *t* statistic for each of these data points. The *t* values are thresholded at an a priori threshold, and the thresholded *t* values are summed across clusters on the basis of spatial (and temporal) adjacency. The significance of the resulting largest cluster’s test statistic is compared to a predefined number of similarly obtained test statistics, after random permutation of the condition labels. Throughout this study, we permuted the values 10.000 times using a *t* test as the test statistic with a threshold of 1.714 (based on 24 participants).

In the interaction analyses, we estimated interactions between the features that were chosen on the basis of the main effects analysis. To foreshadow this, the chosen features were surprisal and bottom-up. All models contained the speech envelope, word onsets, word frequency, and surprisal features. To evaluate the effect of lexical surprisal on the process of structure building, we split the bottom-up feature by the median of surprisal (derived from the trigram model or GPT2). Doing so in one model yielded two separable responses (the model bottomup_split_surprisal from Table 2): a node count TRF for low-surprisal words and a node count TRF for high-surprisal words. We then compared these resulting TRFs using a cluster-based permutation test implemented as spatio_temporal_cluster_test from the MNE-Python library. Any differences between the TRF waveforms can be interpreted as differences in the low-frequency neural readout of structure building between words with low- or high-surprisal values.

**Table 2.** The fitted encoding models in the interaction effects analyses

Model name	Features					
	Envelope / Word onset / Word frequency	Surprisal	Bottom-up / high surprisal	Bottom-up / low surprisal	Bottom-up / random 1	Bottom-up / random 2
bottomup_low_surprisal	X	X		X		
bottomup_high_surprisal	X	X	X			
bottomup_split_surprisal	X	X	X	X		
bottomup_split_random	X	X			X	X

*Note*. X indicates that a feature was included in the model.

Further, to assess the variance explained by the low- versus high-surprisal response to structure7 building, we fit two additional models: a model with only the bottom-up values for high-surprisal words and a model with only the bottom-up values for low-surprisal words. The reconstruction accuracy values from these models were compared to the model main_surprisal from the main effects analysis; this model is identical to those computed here, except for the presence of (half of) the bottom-up node count feature. The difference in reconstruction accuracy between these models—that is, the increase in reconstruction accuracy as a result of the addition of the bottom-up node count feature—was subsequently compared between the low and high surprisal models using a cluster-based permutation test.

In continuation, we wanted to evaluate the reliability of the effects on the TRF waveform (any differences between the low and high surprisal node count TRFs) using the reconstruction accuracy values. Because dichotomizing the node-count feature on the basis of a continuous variable is likely far from the true interaction in the neural signal (the brain probably does not divide words into low or high surprisal categories), a direct comparison of the reconstruction accuracy values from the split feature to an intact feature did not seem a fair comparison. Therefore, we decided to perform evaluation of the effect by comparing the model bottomup_split_surprisal to an equivalent model in which the split was performed randomly (i.e., the words were randomly distributed over two sets).

After obtaining the differences using the cluster-based permutation test and confirming them through the reconstruction accuracy values, we wanted to evaluate whether there was a latency difference between the responses to bottom-up node count for low- or high-surprisal words. To do this, we compared the TRFs for bottom-up node count for the low- and high-surprisal words in a cross-correlation. This cross-correlation was performed on the grand average TRF waveforms of the sensors that were part of the significant clusters resulting from the cluster-based permutation test that compared the two responses. In other words, the sensors were the ones that contributed to the significant difference between the two distributions. We sequentially cross-correlated each sensor and normalized the values by dividing them by the maximal value from the cross-correlation for that sensor. We then obtained the positive peaks for every sensor. The peak corresponds to the “lag” at which the two signals had the highest correlation, and shows how different the two responses are in time. Subsequently, we took the most frequently occurring peak value, and shifted one of the two TRF waveforms to match the other one, and computed the correlation. To check for significance, the same procedure was repeated for randomly selected channels and time-lags 10.000 times.

## RESULTS

### Main Effects: Whole-Brain Averages

Throughout this section, any mention of the features of interest (surprisal, entropy, bottom-up, and top-down) refers to the binomial factor that indicates whether or not this feature was present in the TRF-model as described in Model Fitting and Statistical Analysis, and not to the feature values (e.g., the surprisal values).

**Figure 3.**
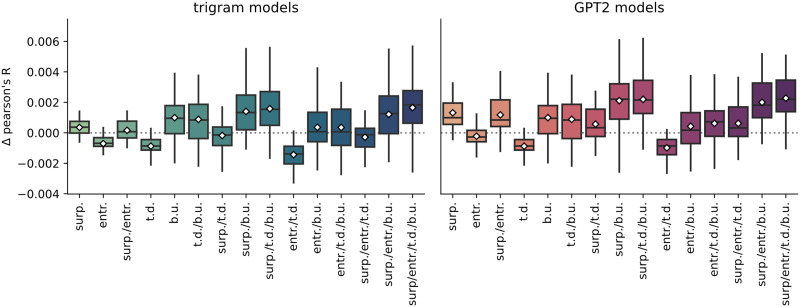
The difference in reconstruction accuracy (delta Pearson’s R) between the base model (speech envelope, word onsets, word frequency) and the other fitted models (see Table 1) for trigram models and GPT2-models. Gray dashed line marks *y* = 0. *Abbreviations*: surp. = surprisal; entr. = entropy; t.d. = top-down; b.u. = bottom-up.

The change in reconstruction accuracy relative to the base model (main_null in Table 1) is shown in Figure 3. The model comparison approach on the whole-brain average reconstruction accuracies of the trigram models (shown left in Figure 3) showed that a model with several interactions between the factors surprisal, entropy, top-down and bottom up was the best descriptor of the data. Specifically, there were interactions between entropy and surprisal, entropy and top-down, surprisal and top-down, and the two syntactic features. The model formula is shown in (1) below. Full model-comparison statistics are provided in Supplementary Materials B, section 1, of the Supporting Information.

(1)  accuracies ∼ entropy + surprisal + topdown + bottomup + entropy * surprisal + entropy * topdown + surprisal * topdown + topdown * bottomup + (1 + topdown * bottomup * surprisal ∣ subject)

The results of this model showed a significant negative effect of entropy (*β* = −6.81 * 10^−4^, *SE* = 5.37 * 10^−5^, *t*(237) = −12.69, *p* < 0.01), indicating that entropy decreased the reconstruction accuracy of the signal. There was a further negative effect of top-down (*β* = −8.88 * 10^−4^, *SE* = 1.51 * 10^−4^, *t*(25.23) = −5.87, *p* < 0.01), similarly suggesting that this feature decreased the reconstruction accuracy of the signal. Bottom-up, on the other hand, had a positive effect on the reconstruction accuracy (*β* = 1.03 * 10^−3^, *SE* = 3.25 * 10^−4^, *t*(22.99) = 3.19, *p* < 0.01), as did surprisal (*β* = 3.65 * 10^−4^, *SE* = 1.31 * 10^−4^, *t*(26.24) = 2.79, *p* < 0.01). In addition, there were several interactions between features. There was an interaction between entropy and surprisal (*β* = 5.02 * 10^−4^, *SE* = 6.20 * 10^−5^, *t*(237) = 8.10, *p* < 0.01) and between top-down and all the other features: entropy (*β* = 1.49 * 10^−4^, *SE* = 6.20 * 10^−5^, *t*(237) = 2.40, *p* < 0.05), surprisal (*β* = 3.45 * 10^−4^, *SE* = 8.33 * 10^−5^, *t*(24.72) = 4.14, *p* < 0.01), and bottom-up (*β* = 7.77 * 10^−4^, *SE* = 7.85 * 10^−5^, *t*(43.24) = 9.98, *p* < 0.01). This suggests that the respective benefit from adding entropy, surprisal, or bottom-up may be affected by the presence of top-down. The full output of the linear mixed model is shown in Table 3.

**Table 3.** Results from the linear mixed effects model evaluating the main effects on trigram models

Factor	Coeff.	Std. Error	*df*	*t* value	*p* value	
(Intercept)	1.14e^−01^	4.70e^−03^	23.08	24.25	<2e^−16^	***
Entropy	−6.81e^−04^	5.37e^−05^	237.00	−12.69	<2e^−16^	***
Surprisal	3.64e^−04^	1.31e^−04^	26.24	2.79	9.70e^−03^	**
Top-down	−8.88e^−04^	1.51e^−04^	25.23	−5.87	3.86e^−06^	***
Bottom-up	1.03e^−03^	3.25e^−04^	22.99	3.19	4.12e^−03^	**
Entropy * Surprisal	5.02e^−04^	6.20e^−05^	237.00	8.10	2.92e^−14^	***
Entropy * Top-down	1.49e^−04^	6.20e^−05^	237.00	2.40	0.02	*
Surprisal * Top-down	3.45e^−04^	8.33e^−05^	24.72	4.15	3.47e^−04^	***
Top-down * Bottom-up	7.77e^−04^	7.85e^−05^	43.24	9.89	1.12e^−12^	***

*Note*. Signif. codes: 0 ‘***’  0.001 ‘**’  0.01 ‘*’  0.05 ‘.’  0.1 ‘ ’.

The model comparison approach on the whole-brain average reconstruction accuracies of the GPT2 models (see Figure 5 in the next section) revealed a similar pattern. Indeed, the same model fit best to the data. The full model is displayed in (2) below. Again, model comparison statistics are provided in Supplementary Materials B, section 2.

(2)  accuracies ∼ entropy + surprisal + topdown + bottomup + entropy * surprisal + entropy * topdown + surprisal * topdown + topdown * bottomup + (topdown * bottomup * surprisal ∣ subject)

Using GPT2, there was also positive effect of surprisal (*β* = 1.20 * 10^−3^, *SE* = 6.63 * 10^−5^, *t*(28.20) = 6.46, *p* < 0.01) and of bottom-up (*β* = 7.95 * 10^−4^, *SE* = 3.03 * 10^−4^, *t*(23.82) = 2.63, *p* < 0.05). In addition, top-down significantly decreased average reconstruction accuracy of the signal (*β* = −9.03 * 10^−4^, *SE* = 1.37 * 10^−4^, *t*(39.23) = −6.61, *p* < 0.01), as did entropy (*β* = −3.89 * 10^−4^, *SE* = 6.63 * 10^−5^, *t*(282.80) = −5.87, *p* < 0.01). Here, too, there were interactions between surprisal and entropy (*β* = −2.61 * 10^−4^, *SE* = 7.65 * 10^−5^, *t*(282.80) = 3.41, *p* < 0.01), and between top-down and the other features (entropy: *β* = 1.97 * 10^−4^, *SE* = 7.65 * 10^−5^, *t*(282.80) = 2.57, *p* < 0.05; surprisal: *β* = 1.53 * 10^−4^, *SE* = 7.65 * 10^−5^, *t*(282.80) = 1.99, *p* < 0.05; bottom-up: *β* = 8.43 * 10^−4^, *SE* = 7.65 * 10^−5^, *t*(282.80) = 11.01, *p* < 0.01), suggesting that the effect of top-down may be less negative when bottom-up is part of the model. The full output of the linear mixed model is displayed in Table 4.

**Table 4.** Results from the linear mixed effects model evaluating the main effects on GPT2 models

Factor	Coeff.	Std. error	*df*	*t* value	*p* value	
(Intercept)	1.14e^−01^	4.63e^−03^	23.14	24.45	<2e^−16^	***
Entropy	−3.89e^−04^	6.63e^−05^	282.80	−5.87	1.22e^−08^	***
Surprisal	1.20e^−03^	1.86e^−04^	28.20	6.46	5.25e^−07^	***
Top-down	−9.03e^−04^	1.37e^−04^	39.23	−6.61	7.31e^−08^	***
Bottom-up	7.95e^−04^	3.03e^−04^	23.82	2.63	0.01	*
Entropy * Surprisal	2.61e^−04^	7.65e^−05^	282.80	3.41	7.54e^−04^	***
Entropy * Top-down	1.97e^−04^	7.65e^−05^	282.80	2.57	0.01	*
Surprisal * Top-down	1.53e^−04^	7.65e^−05^	282.80	1.99	0.05	*
Top-down * Bottom-up	8.43e^−04^	7.65e^−05^	282.80	11.01	<2e^−16^	***

*Note*. Signif. codes: 0 ‘***’  0.001 ‘**’  0.01 ‘*’  0.05 ‘.’  0.1 ‘ ’.

To assess any differences between the trigram and GPT2 estimates of surprisal and entropy, we selected the models that included only these estimates (the top four models from Table 1: main_null, main_entropy, main_surprisal, and main_distributional) and subjected the average reconstruction accuracy for each participant, model, and language model to a linear mixed model with the factors entropy, surprisal, language model (trigram or GPT2), and their interactions. We performed model comparison on fixed and random effects in the same way as described above. Model comparison statistics are provided in Supplementary Materials B, section 3.

The best model had the following structure:

(3)  accuracies ∼ model * entropy * surprisal + (1 + model * surprisal ∣ subject)

This model revealed the same effects on surprisal and entropy as shown above; a positive effect of surprisal (*β* = 1.32 * 10^−3^, *SE* = 1.10 * 10^−4^, *t*(28.08) = 5.53, *p* < 0.01) and a negative effect of entropy (*β* = −2.22 * 10^−4^, *SE* = 1.04 * 10^−6^, *t*(115) = −2.15, *p* < 0.05). In addition, there was an interaction between language model and surprisal (*β* = 9.61 * 10^−4^, *SE* = 2.35 * 10^−4^, *t*(35.76) = 4.09, *p* < 0.01), suggesting that the increase of reconstruction accuracy as a result of surprisal was larger in the GPT2 models than in the trigram models, and an interaction between language model and entropy (*β* = 4.85 * 10^−4^, *SE* = 1.47 * 10^−4^, *t*(115.00) = 3.31, *p* < 0.01), indicating that the effect of entropy was more negative for the trigram models than the GPT2 models. Finally, there was a three-way interaction between language model, entropy, and surprisal (*β* = 4.28 * 10^−4^, *SE* = 2.07 * 10^−4^, *t*(115.00) = 2.07, *p* < 0.05). Post-hoc comparisons revealed that this was because the interaction between entropy and surprisal was significant for the trigram models, but not for the GPT2 models (GPT2 models: *F*(1, 92) = 0.35, *p*_Bonferroni_ = 1; trigram models: *F*(1, 92) = 12.34, *p*_Bonferroni_ < 0.01). The interaction between entropy and surprisal in the trigram models was driven by an effect for entropy only when surprisal was not in the model (no surprisal: *F*(1, 92) = 46.59, *p*_Bonferroni_ < 0.01; surprisal: *F*(1, 92) = 3.45, *p*_Bonferroni_ = 0.27). The full model output is shown in Table 5.

**Table 5.** Results from the linear mixed effects model comparing GPT and trigram models

Factor	Coeff.	Std. error	*df*	*t* value	*p* value	
(Intercept)	1.14e^−01^	4.86e^−03^	23.01	23.38	<2e^−16^	***
Language model	−6.58e^−15^	1.10e^−04^	85.52	0.00	1.00	
Entropy	−2.22e^−04^	1.04e^−06^	115.00	−2.15	0.03	*
Surprisal	1.32e^−03^	2.38e^−04^	28.08	5.53	6.55e^−06^	***
Language model * entropy	−4.85e^−04^	1.47e^−04^	115.00	−3.31	1.25e^−03^	**
Language model * surprisal	−9.61e^−04^	2.35e^−04^	35.76	−4.09	2.33e^−04^	***
Entropy * surprisal	8.61e^−05^	1.47e^−04^	115.00	0.59	0.56	
Language model * entropy * surprisal	4.28e^−04^	2.07e^−04^	115.00	2.07	0.04	*

*Note*. Signif. codes: 0 ‘***’  0.001 ‘**’  0.01 ‘*’  0.05 ‘.’  0.1 ‘ ’.

In summary, the features surprisal and bottom-up had positive effects on the whole-brain average reconstruction accuracy. The features entropy and top-down appear to bring down the whole-brain average reconstruction accuracy values. In addition, the presence of the top-down feature affects the relative benefit (or detriment) of the other features. Furthermore, the GPT2-derived surprisal features are a better predictor for the delta-band neural signal than the trigram-derived surprisal features.

### Main Effects: Cluster-Based Permutation Tests

**Figure 4.**
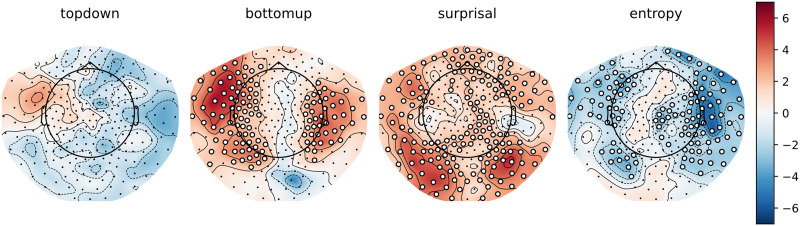
Trigram models. Scalp maps of the *t* values resulting from the contrast between the averages of all models that contain a specific predictor (e.g., top-down) and all models that do not contain this predictor. Each scalp map represents this contrast for a different feature. White dots on the scalp map indicate the sensors that contributed to the clusters that allowed us to reject the null hypothesis (i.e., the difference is not 0).

**Figure 5.**
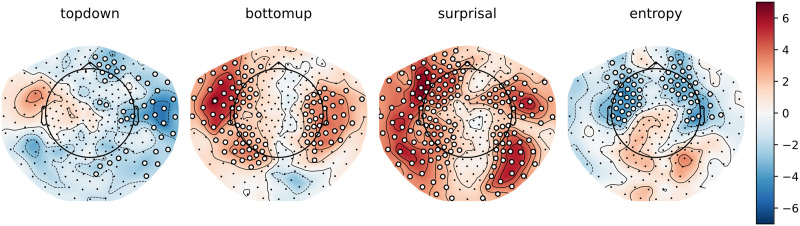
GPT2 models. Scalp maps of the *t* values resulting from the contrast between the averages of all models that contain a specific predictor (e.g., top-down) and all models that do not contain this predictor. Each scalp map represents this contrast for a different feature. White dots on the scalp map indicate the sensors that contributed to the clusters that allowed us to reject the null hypothesis (i.e., the difference is not 0).

To gain some insight into the spatial distribution of the effects described above, we computed the per-participant, per-sensor averages for the models that did or did not include a given feature. As can be observed in Figure 4 and Figure 5 below, the general pattern is the same as in the analysis on the whole-brain average reconstruction accuracies: bottom-up and surprisal contribute positively to model fit, while entropy and top-down do not. Both bottom-up node counts and surprisal values contribute to model fit in large, bilateral clusters, although the bottom-up node count feature notably contributes more to model fit in the left hemisphere. As for surprisal, it is clear that both GPT2-derived and trigram surprisal values contribute to model fit in almost all sensors. The pattern of improvement is slightly different between the two, with less contribution around auditory areas from the trigram surprisal values. The difference was not tested statistically, so we do not draw conclusions on the basis of this. Both measures of entropy appear to decrease model fit bilaterally. The top-down node count feature is a curious case: there is no evidence for an improvement, but the decrease is significant only in the right hemisphere when the additional features are drawn from GPT2 models.

With respect to our first question, these analyses revealed that as a general predictor of the delta-band neural signal, surprisal estimates from large models like GPT2 outperform the short-context trigram models. In addition, from a methodological perspective, the above analyses clearly show the non-trivial effect the addition of some features to the TRF model can have on the reconstruction accuracy of other features using ridge regression specifically, even when these features are not dangerously correlated as indicated by the VIF. For the purposes of the rest of the present study, these analyses have provided sufficient evidence for the positive contributions of bottom-up node count and lexical surprisal features in a model of the delta-band neural signal. We will therefore continue further analyses on these two features, and remove top-down node count and lexical entropy from our models.

### Interaction Effects

To investigate whether the process of syntactic structure building is affected by lexical distributional information, we split the bottom-up node count feature into two features: bottom-up node count for low-surprisal words (i.e., words that are statistically relatively predictable from the context) and high-surprisal words (i.e., words that are statistically relatively unpredictable from the context). We then compared the resulting TRFs, which capture the neural response to bottom-up structure building, between these surprisal conditions. Differences between the TRFs provide insight into how the process of structure building may be mediated by distributional information.

#### Trigram models

**Figure 6.**
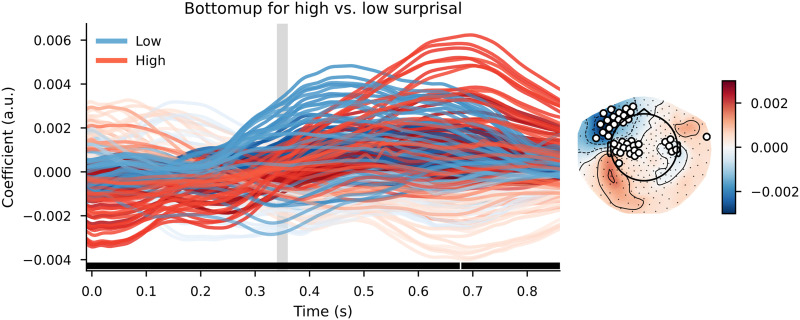
The bottom-up node count temporal response function (TRF) for high surprisal (in red) and low surprisal (in blue) with surprisal from the trigram model as the dividing estimate. Individual lines represent sensors. The displayed sensors contributed to the clusters that allowed us to reject the null hypothesis. Black bars indicate time points that contributed to clusters that allowed us to reject the null hypothesis. Vertical gray bar is the time point of the scalp map displayed on the right. The scalp map shows the difference between the coefficients from the high- and low-surprisal words. White dots on the scalp map indicate the sensors that contributed to the clusters that allowed us to reject the null hypothesis at the time point of the gray bar.

When using a simple trigram model for surprisal estimation and using those values to divide the words over high surprisal and low surprisal categories, the cluster-based permutation test reveals a widespread difference between the TRFs. The clusters that contributed to the difference between the distributions had a bilateral distribution across the scalp, although the effects were most pronounced in the left-frontal area. The clusters were spread out across the entire time window, meaning that the effects were visible slightly before word onset and lasted approximately 1 s after word onset. As can be observed in Figure 6, the response to bottom-up node count appears to be slowed down and increased in magnitude in high-surprisal words relative to the low-surprisal words.

**Figure 7.**
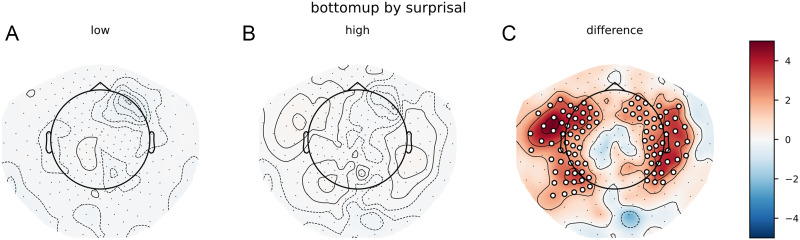
Trigram models. Scalp maps of the *t* values resulting from the contrast between the model main_surprisal and the model with (A) only low surprisal bottom-up node counts or (B) high surprisal bottom-up node counts. (C) The contrast between those contrasts, that is, the difference in increase of reconstruction accuracy between high and low surprisal bottom-up node counts. Red values show that the high surprisal bottom-up node counts explain more variance. White dots on the scalp map indicate the sensors that contributed to the clusters that allowed us to reject the null hypothesis (i.e., the difference is not 0).

The pattern on the reconstruction accuracy values was slightly different. Despite this large difference between the bottom-up node count response to high- and low-surprisal words, the cluster-based permutation test revealed no difference between a model that split the bottom-up node count feature by surprisal and a model that split the bottom-up node count feature randomly. In addition, a comparison between the relative benefit of the high- and low-surprisal bottom-up node count features revealed that the high surprisal bottom-up node count feature explained more variance in the delta-band neural signal than did the low-surprisal feature (see Figure 7C). Comparing the effects of these halves of the bottom-up feature separately (i.e., a model with only the high surprisal bottom-up feature vs. a model that did not include this feature or a model with only the low surprisal bottom-up feature vs. a model that did not include this feature) did not reveal any effects (Figure 7A and B).

#### GPT2 models

**Figure 8.**
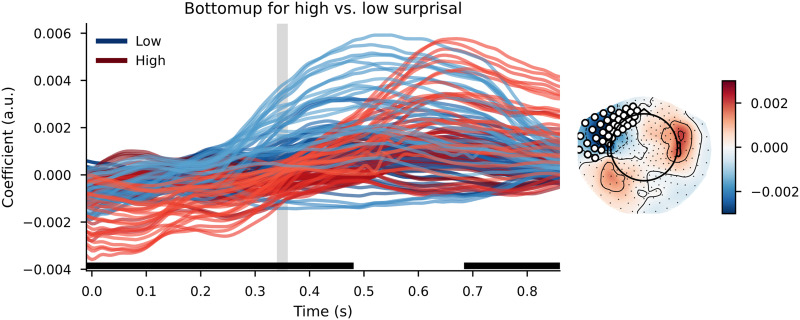
The bottom-up node count TRF for high surprisal (in red) and low surprisal (in blue) with surprisal from GPT2 as the dividing estimate. Individual lines represent sensors. The displayed sensors contributed to the clusters that allowed us to reject the null hypothesis. Black bars indicate time points that contributed to clusters that allowed us to reject the null hypothesis. Vertical gray bar is the time point of the scalp map displayed on the right. The scalp map shows the difference between the coefficients from the high- and low-surprisal words. White dots on the scalp map indicate the sensors that contributed to the clusters that allowed us to reject the null hypothesis at the time point of the gray bar.

When using a complex language model (GPT2) to divide words over high surprisal and low surprisal categories, the general pattern on the TRFs was similar. The cluster-based permutation test revealed a widespread difference between the bottom-up node count responses for low-surprisal and high-surprisal words. The clusters that contributed to this difference had a wide temporal distribution, with clusters between before word onset to 500 ms after word onset and a cluster at the end of the time window—approximately from 700 ms onwards. Again, the most prominent difference was in left temporal/frontal sensors. Visual inspection of the TRFs (Figure 8) suggests, again, a later response to the bottom-up node count feature for high-surprisal words than for low-surprisal words.

The pattern on the reconstruction accuracies was inconclusive. The cluster-based permutation test comparing the reconstruction accuracy values for models with a random split of the bottom-up node feature to the model with a surprisal-based split of the bottom-up node count feature revealed no effects. There were also no effects for the separate node-count predictors on the reconstruction of the neural signal relative to a base model, nor was there a difference between these differences.

Taken together, the results from the interaction analyses suggest that even a short-context surprisal value affects the timing of structure building (in a bottom-up fashion). The lack of effects on the reconstruction accuracy values make the results difficult to interpret. Apparently, a systematic split for node count by surprisal does not lead to a significantly higher reconstruction accuracy than a random split for node count. This suggests that the effect is small—or there may be confounding factors that obscure the state of affairs.

### Correction for Word Duration

Indeed, there are several factors that potentially correlate with (or are causal of) surprisal. One of the correlating factors that is not in itself causal of surprisal values in a way that, for example, word frequency might be, is particularly interesting for our current finding: the factor of word duration. Indeed, higher surprisal values tend to be associated with longer word durations (Mahowald et al., 2013; Piantadosi et al., 2011); this is also the case in our data (trigram models: duration of high-surprisal words is significantly higher than the duration of low-surprisal word; *t*(8548) = 49.14, *p* < 0.01, mean_high_ (*SD*) = 0.32 s (0.16); mean_low_ (*SD*) = 0.17 s (0.12); significant positive correlation between duration and surprisal (*ρ* = 0.59; *p* < 0.01); GPT2 models: duration of high-surprisal words is significantly higher than the duration of low-surprisal word; *t*(8548) = 37.55, *p* < 0.01, mean_high_ (*SD*) = 0.31 s (0.17); mean_low_ (*SD*) = 0.19 s (0.13); significant positive correlation between duration and surprisal (*ρ* = 0.42; *p* < 0.01). This suggests that the later response to node counts could—very simply—be an effect of slower processing of longer words (New et al., 2006; Tyler et al., 2000).

**Figure 9.**
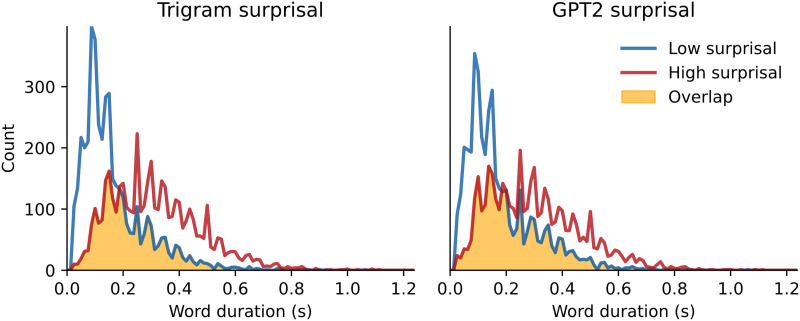
The histograms of word duration (100 bins between 0.0 and 1.25 s) split along the median of surprisal (low surprisal in blue, high surprisal in red). The yellow shaded area is the overlapping distribution from which words were selected to correct for differences in word duration.

To examine whether word duration could explain our effects, we extracted the TRFs for bottom-up node count only for words that were matched for duration. We did this by computing a histogram (100 bins) for word duration in both low and high surprisal conditions, and extracting the overlap between these two histograms. The distributions and their overlap are displayed in Figure 9. To extract (an approximation of) the overlap between two distributions, we calculated how many words should be contained in each bin to form two identical histograms. This boiled down to picking the lowest number of words from the two histograms in a given bin. For example, if we consider a bin that contains words with duration 0.20–0.21 s in which there are 100 words in the high surprisal group and 150 in the low surprisal group, the value in our overlapping histogram should be 100. We then took a random subsample of words from both the low and high surprisal conditions in each bin, such that the resulting distributions of word durations for low- and high-surprisal words were indistinguishable (the yellow shading in Figure 9). This resulted in a subset of approximately half of the words (4,556 out of 8,550 words). All words selected in this analysis came from the yellow shaded distribution, creating two sets of words that differed in their surprisal value along the median, but that had near-identical distributions of word duration. We then used only these words to compute the bottom-up TRF in low and high surprisal conditions, as well as a random split (like above).

#### Trigram models

**Figure 10.**
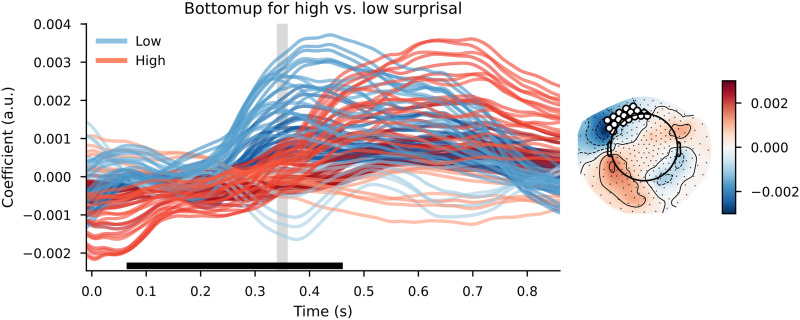
The bottom-up node count TRF for high surprisal (in red) and low surprisal (in blue) with surprisal from the trigram model as the dividing estimate, after correction for word duration. Individual lines represent sensors. The displayed sensors contributed to the clusters that allowed us to reject the null hypothesis. Black bars indicate time points that contributed to clusters that allowed us to reject the null hypothesis. Vertical gray bar is the time point of the scalp map displayed on the right. The scalp map shows the difference between the coefficients from the high- and low-surprisal words. White dots on the scalp map indicate the sensors that contributed to the clusters that allowed us to reject the null hypothesis at the time point of the gray bar.

Despite this extensive subsampling, the cluster-based permutation test revealed a significant difference between the responses to bottom-up node count. The clusters that contributed to this difference had a left frontotemporal distribution and were constrained to a relatively early time window—between 50 and 450 ms. Visual inspection of the waveforms again suggested a temporal shift in the response to bottom-up node count, with the bottom-up node count response delayed for high-surprisal words relative to low-surprisal words (see Figure 10).

**Figure 11.**
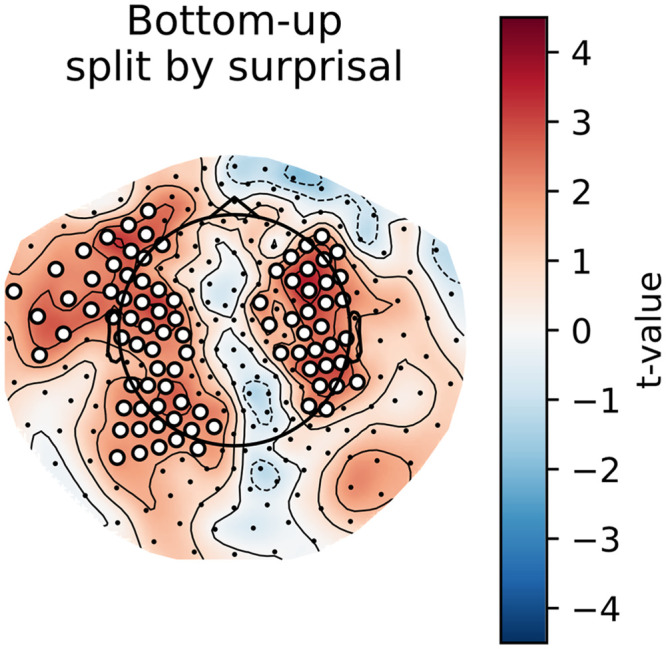
Scalp map of the *t* values resulting from the contrast: systematic split of the bottom-up predictor vs. random split of the bottom-up predictor, using surprisal from the trigram model as the dividing estimate. White dots on the scalp map indicate the sensors that contributed to the clusters that allowed us to reject the null hypothesis (i.e., the difference is not 0).

The assessment of significance through the reconstruction accuracy values showed a clearer image this time: the cluster-based permutation test that assessed difference between a systematic split by surprisal and a random split of the bottom-up node count values came back significant, with reconstruction accuracies higher when the bottom-up node count values were split by the median surprisal values than when they were split randomly (see Figure 11). Despite this difference being significant, there were no differences between the contribution of the bottom-up node count feature for low- or high-surprisal words. This difference did exist before controlling for word duration (see Trigram Models subsection of The Role of Word Recognition).

The significantly different reconstruction accuracy values between models with randomly split bottom-up predictors and surprisal split bottom-up predictors confirms the effects found on the TRF waveforms. A model that allows for variation between words on the basis of surprisal leads to a better description of the signal than a model that allows variation randomly. This suggests that the temporal shift we observe visually is indeed there. To look into this in more detail, we computed the cross-correlation between the high and low surprisal bottom-up node count responses for the sensors that contributed to the difference between the two distributions.

**Figure 12.**
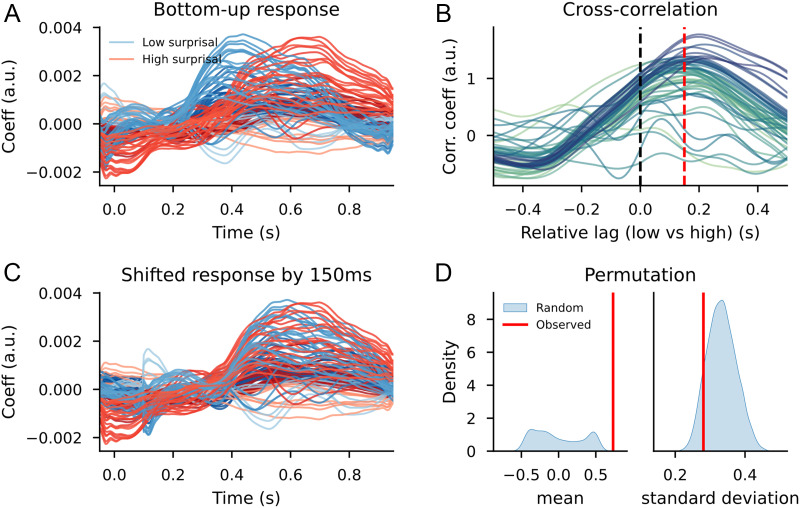
Cross-correlation results for the trigram models, after selection for word duration. (A) Bottom-up TRF time courses for the sensors from the cluster-based permutation test between high surprisal (in red) and low surprisal (in blue). (B) Cross-correlation between the high and low surprisal bottom-up responses for the sensors from the clusters (scaled). Colors indicate sensors. (C) The shifted response from the low surprisal condition (in blue) to overlap with the high surprisal condition (in red). (D) Kernel density plots of means and standard deviations from correlations between randomly selected sensors at shifted randomly selected lags; the red bar indicates the values observed from the sensors selected after the cluster-based permutation test shifted at the lags from the cross-correlation.

The cross-correlation on the sensors that were part of the clusters (depicted in Figure 10) revealed that the time point at which the correlation was highest for most sensors was at 150 ms post word onset. The average correlation between the shifted low surprisal (as shown in Figure 12C) and the original high surprisal response was 0.73 (*SD* = 0.28), with a maximum of 0.95. This high correlation for a large subset of channels, which did not exist for random selections of channels and time shifts (see Figure 12D) suggests that the response to high-surprisal words was delayed by 250 ms relative to the low-surprisal words. At the same time, the relatively high variance between channels may indicate either that the temporal shift is not uniform (i.e., not all readouts of structure building (potentially with different neural sources) are temporally affected by contextual surprisal) or that some readouts of structure building from different neural sources respond qualitatively different as a function of surprisal (i.e., shifting them in time in any direction will not increase the correlation).

#### GPT2 models

**Figure 13.**
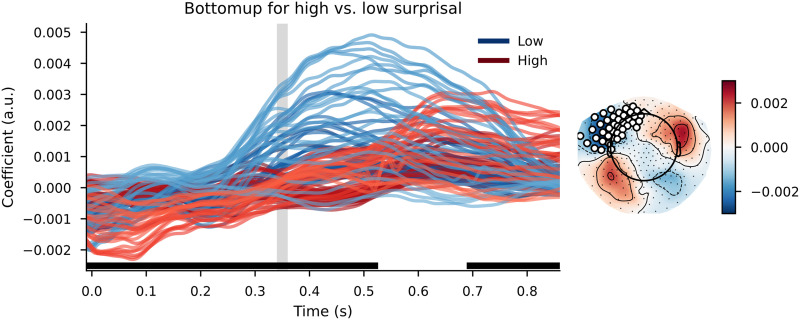
The bottom-up node count TRF for high surprisal (in red) and low surprisal (in blue) with surprisal from GPT2 as the dividing estimate, after correction for word duration. Individual lines represent sensors. The displayed sensors contributed to the clusters that allowed us to reject the null hypothesis. Black bars indicate time points that contributed to clusters that allowed us to reject the null hypothesis. Vertical gray bar is the time point of the scalp map displayed on the right. The scalp map shows the difference between the coefficients from the high- and low-surprisal words. White dots on the scalp map indicate the sensors that contributed to the clusters that allowed us to reject the null hypothesis at the time point of the gray bar.

**Figure 14.**
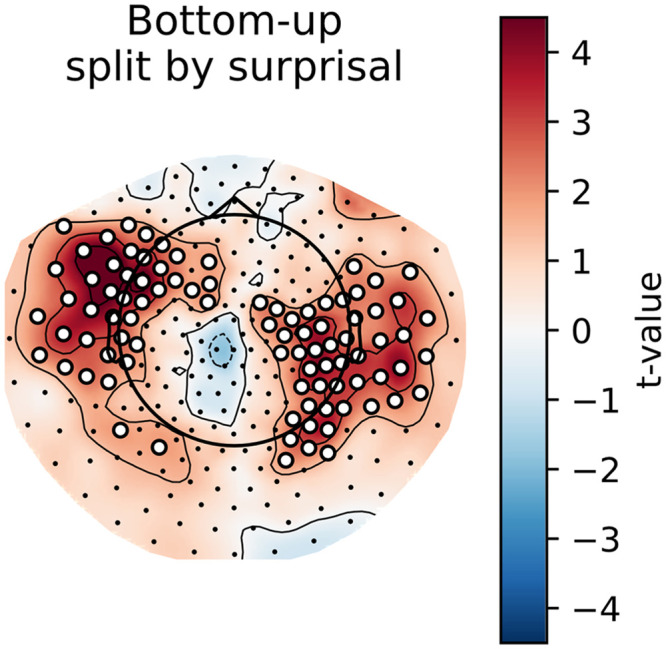
Scalp map of the *t* values resulting from the contrast: systematic split of the bottom-up predictor vs. random split of the bottom-up predictor, using surprisal from GPT2 as the dividing estimate. White dots on the scalp map indicate the sensors that contributed to the clusters that allowed us to reject the null hypothesis (i.e., the difference is not 0).

Despite the subsampling, the difference between high and low surprisal bottom-up node count responses remained when the split was based on GPT2-extracted surprisal values. The cluster-based permutation test showed a difference between the two time courses. Clusters that contributed to this difference were once again prominent in left-frontotemporal areas and spanned a wide time window, with one cluster ranging from word onset to approximately 550 ms, and a second cluster in a late time window from around 700 ms onwards (Figure 13). As in the analysis on the split by trigram surprisal, excluding words on the basis of an extremely long or short duration clarified the picture; splitting the bottom-up node count feature on the basis of surprisal led to higher reconstruction accuracy than splitting the bottom-up node count feature randomly (Figure 14).

**Figure 15.**
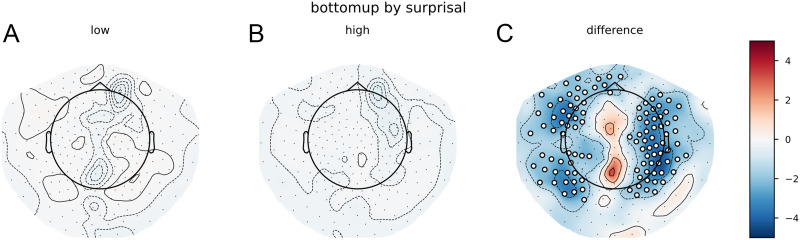
GPT2 models after correction for word duration. Scalp maps of the *t* values resulting from the contrast between the model main_surprisal and the model with (A) only low surprisal bottom-up node counts or (B) high surprisal bottom-up node counts. (C) The contrast between those contrasts, i.e. the difference in increase of reconstruction accuracy between high and low surprisal bottom-up node counts. Red values show that the high surprisal bottom-up node counts explain more variance. White dots on the scalp map indicate the sensors that contributed to the clusters that allowed us to reject the null hypothesis (i.e., the difference is not 0).

While there was no difference between the high and low-surprisal bottom-up node count features when it comes to variance explained before correcting for word duration, there was a difference now: Low surprisal bottom-up node counts explained more variance than the high surprisal counterpart (Figure 15). This is in stark contrast with the findings on the trigram models, where we observed a larger contribution to the reconstruction accuracy for the high surprisal bottom-up node counts that disappeared after correcting for word duration.

**Figure 16.**
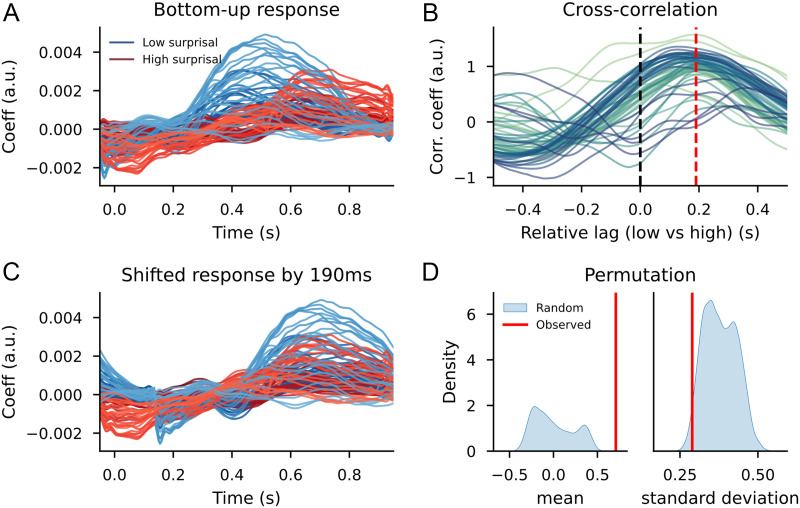
Cross-correlation results for the GPT2 models, after selection for word duration. (A) Bottom-up TRF time courses for the sensors from the cluster-based permutation test between high surprisal (in red) and low surprisal (in blue). (B) Cross-correlation between the high and low surprisal bottom-up responses for the sensors from the clusters (scaled). Colors indicate sensors. (C) The shifted response from the low surprisal condition (in blue) to overlap with the high surprisal condition (in red). (D) Kernel density plots of means and standard deviations from correlations between randomly selected sensors at shifted randomly selected lags; the red bar indicates the values observed from the sensors selected after the cluster-based permutation test shifted at the lags from the cross-correlation.

To further investigate the potential temporal shift of the response as a function of surprisal, we performed a cross-correlation analysis. The cross-correlation revealed that the time point at which the correlation was highest for most sensors was at 190 ms post word onset. The correlation was 0.71 on average (*SD* = 0.29), with a maximum of 0.93. As before, the high correlation, which did not exist for a random subset of channels and time points (see Figure 16D), suggests that the response indeed varies in time as a function of lexical surprisal. Again, relatively large variance suggests that this may not be the case for all sensors in the selection—the temporal shift may not be the same for all sensors, or the response qualitatively differs between low- and high-surprisal words for some sensors.

When we jointly consider all of the results above, a pattern emerges. First, there is a clear indicator that temporal properties of a readout of structure building, extracted with bottom-up node counts, are affected by lexical surprisal; structure building operations are performed later when the surprisal of the word to be integrated in the sentence is higher relative to when the surprisal of that word is lower. When we consider a split by the median, this temporal shift appears quite large: between 150 and 200 ms. This is confirmed by the reconstruction accuracy values; reconstruction accuracy values are higher when the bottom-up node count feature is split systematically using surprisal than when the feature is spit randomly.

Second, when it comes to the amount of variance explained by structure-building operations, the pattern is affected by word duration. When we do not correct for word duration, we observe a larger variance explained by bottom-up node count for high surprisal than for low surprisal, but only when surprisal is extracted from a trigram model; there is no difference when surprisal is extracted from GPT2. However, after correction for word duration, we observe that the difference found for the trigram models disappears—that is, high surprisal bottom-up node count no longer explains more variance than low surprisal bottom-up node count, and the reverse pattern is seen for the analysis using GPT2. Here, low surprisal bottom-up node counts appear to explain more variance than the high surprisal bottom-up node counts.

These observations pattern with the amplitudes of the TRF waveforms. In the trigram model, before correcting for word duration, the amplitude of the node count-response to high-surprisal words is larger than the amplitude of the response to low-surprisal words (see Figure 6). This difference appears to disappear after correction for word duration (see Figure 10). At the same time, there is no obvious amplitude difference between the high and low surprisal node-count responses from the GPT2 model (Figure 8), while the low surprisal bottom-up node count has an obviously larger amplitude after correction for word duration (Figure 13). Taken together, this suggests a nontrivial relationship between word duration, language model for surprisal estimation, and response amplitude. We will return to this in the discussion.

### The Role of Word Recognition

A 150 to 190 ms shift in response time begs the question, To what extent is the delay driven by lexical contextual information directly affecting structure-building operations? After all, there is an important process that—in an interactive, cascaded model of language comprehension—occurs prior to or simultaneously with the generation of syntactic structure: word recognition. A word that is predictable from the context is recognized faster (Grosjean & Itzler, 1984) and read faster (Amenta et al., 2023; Aurnhammer & Frank, 2019). In a cascaded architecture, then, an earlier completion or faster process of word recognition could affect the time course of the inference of syntactic structure.

To investigate this, we performed the same contrast for high versus low surprisal on a feature that captures the presence of lexical information in the neural signal: word frequency (Slaats et al., 2023). That is, this time, we split the word frequency feature into two separate features on the basis of the surprisal values (high-surprisal-word frequency, low-surprisal-word frequency). Again, we only performed this analysis for the words obtained from the overlapping distributions of word length to exclude the possibility that word duration drives any of the effects.

#### Trigram models

**Figure 17.**
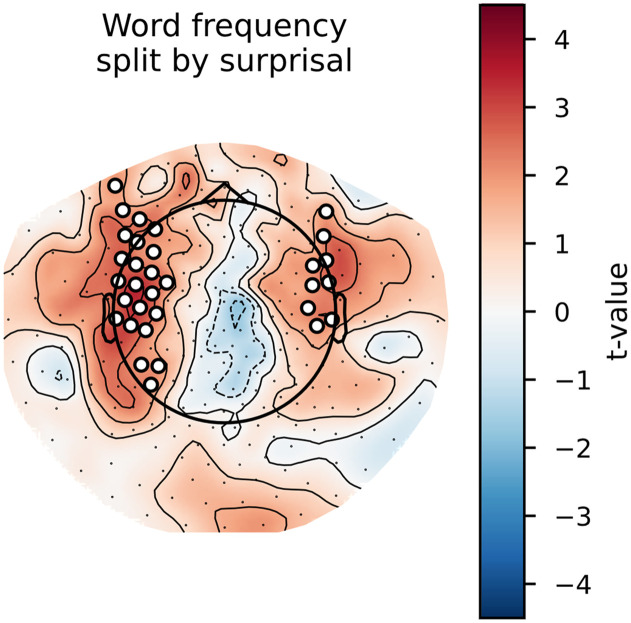
Scalp map of the *t* values resulting from the contrast: systematic split of the word frequency predictor vs. random split of the word frequency predictor, using surprisal from GPT2 as the dividing estimate. White dots on the scalp map indicate the sensors that contributed to the clusters that allowed us to reject the null hypothesis (i.e., the difference is not 0).

**Figure 18.**
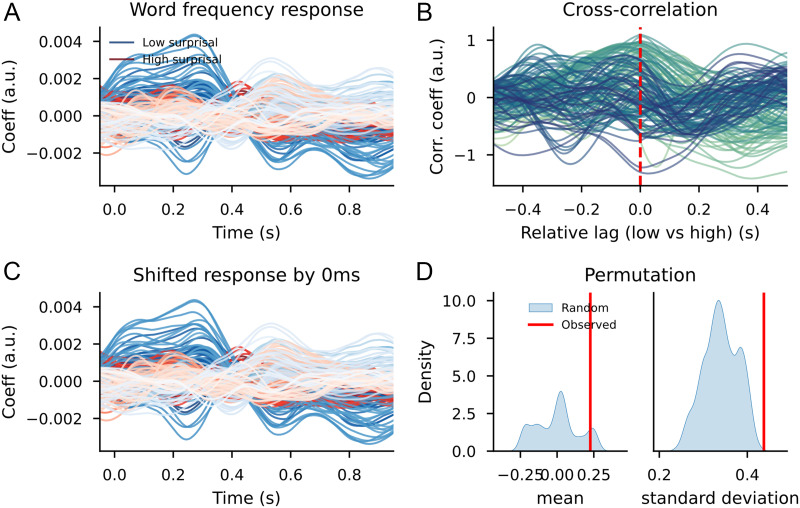
Cross-correlation results for the trigram models on the word frequency feature, after selection for word duration. (A) Word frequency TRF time courses for the sensors from the cluster-based permutation test between high surprisal (in red) and low surprisal (in blue). (B) Cross-correlation between the high- and low-surprisal-word frequency responses for the sensors from the clusters (scaled). Colors indicate sensors. (C) The shifted response from the low surprisal condition (in blue) to overlap with the high surprisal condition (in red). (D) Kernel density plots of means and standard deviations from correlations between randomly selected sensors at shifted randomly selected lags; the red bar indicates the values observed from the sensors selected after the cluster-based permutation test shifted at the lags from the cross-correlation.

Interestingly, the cluster-based permutation test revealed that the word frequency response differed between high- and low-surprisal words, suggesting that there is indeed a difference in lexical processing between high- and low-surprisal words. This is further confirmed by a higher reconstruction accuracy for a split of word frequency by surprisal than a random split of word frequency (see Figure 17). However, this difference is crucially *not* temporal in nature. In fact, it appears to be one of amplitude; coefficients are higher for the low-surprisal words than for the high-surprisal words. The cross-correlation on the sensors that differed between conditions revealed that the correlation between the word frequency response to high- and low-surprisal words was highest at a delay of 0 ms. This indicates that there is no detectable time shift, which can be clearly observed in Figure 18A–C. The correlation between the two responses was not high on average (mean = 0.22), though there was considerable variance: with a standard deviation of 0.44, some channels had a Pearson’s correlation coefficient of 0.97 between the two conditions, though more than 45% of the channels had a correlation coefficient lower than 0.2.

#### GPT2 models

**Figure 19.**
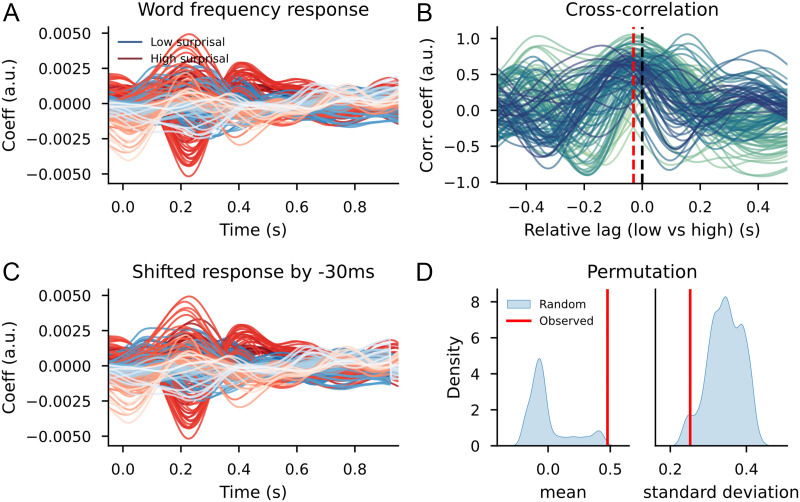
Cross-correlation results for the GPT2 models on the word frequency feature, after selection for word duration. (A) Word frequency TRF time courses for the sensors from the cluster-based permutation test between high surprisal (in red) and low surprisal (in blue). (B) Cross-correlation between the high- and low-surprisal-word frequency responses for the sensors from the clusters (scaled). Colors indicate sensors. (C) The shifted response from the low surprisal condition (in blue) to overlap with the high surprisal condition (in red). (D) Kernel density plots of means and standard deviations from correlations between randomly selected sensors at shifted randomly selected lags; the red bar indicates the values observed from the sensors selected after the cluster-based permutation test shifted at the lags from the cross-correlation.

When using GPT2 to divide words over high and low surprisal condition, we again observe a difference between the responses that appear mostly of amplitude (see Figure 19A). However, the difference between a GPT2 split and a random split does not reach significance. If we use the sensors that are part of clusters that contribute to the significant difference between the two responses to perform a cross-correlation, we observe a similar pattern as for the trigram model. The highest correlation between the two responses was at a time lag close to zero; the two responses were most similar at a delay of −30 ms—which positions the response to high-surprisal words slightly before the response to low-surprisal words. In other words, despite there being a timing effect here, this effect is in the opposite direction. These analyses suggest therefore that temporal differences in the process of lexical retrieval are not the cause of the delayed response to bottom-up structure building—though qualitative differences between the processes can still play a role.

## DISCUSSION

In this study, we investigated how the delta-band neural signal represents and exploits lexical distributional information in the process of syntactic structure building during auditory language comprehension. We approached this question in two main subquestions. First, we asked whether trigram- or GPT2-derived estimates of lexical surprisal are a better model of the delta-band neural signal during language comprehension. Second, we asked whether the delta-band neural readout of syntactic structure building changes as a function of the distributional properties of a word, and if this influence can be linked to probabilities based on the immediately preceding words (as reflected in surprisal and entropy estimates from a trigram model), or rather to the larger context (as reflected in GPT2 models).

To answer these questions, we used a modeling approach and a naturalistic listening paradigm. We presented participants with audiobooks while we recorded their MEG, and analyzed the resulting data using TRFs. This linear regression approach allowed us to study high-level processes during language comprehension, while controlling for lower level processes like speech tracking. Our analysis consisted of two parts: a main-effects analysis to evaluate which features modeled the delta-band neural signal most accurately; and an interaction analysis, to evaluate whether lexical distributional information affects the process of syntactic structure building (the inference of syntactic structure).

### Describing the Delta-Band Neural Signal: Surprisal and Bottom-Up Node Counts

The main effects analysis showed that the features that contributed positively to models of the data were bottom-up node counts and lexical surprisal. These features were used for further analysis in the interaction analysis. This finding is in accordance with studies that used other features, such as phonemes, to model the delta-band neural signal: the delta-band signal is modeled better with surprisal than with entropy, while the opposite is true for the theta band (4–8/10 Hz; Donhauser & Baillet, 2020; Mai & Wang, 2023). That the delta-band neural signal is influenced by syntactic structure is known (Kaufeld et al., 2020; Lo et al., 2022; Lu et al., 2022; Slaats et al., 2023), but which parsing strategy yields the best predictors for the delta-band neural signal is an open question. Some studies suggest that bottom-up parsing strategies are more predictive of the neural signal (Giglio et al., 2024; Nelson, El Karoui, et al., 2017), others found no difference (Brennan et al., 2016), and there is even evidence for importance of the top-down strategy in favor of bottom-up (Coopmans, 2023). It is likely that the exact paradigm (production, comprehension) methodology (EEG, fMRI, iEEG, MEG) and analysis choices (source localization, TRF-estimation algorithm, etc.) influence the outcome of this comparison. In our study, bottom-up node counts had good performance, and for this reason, we continued with this feature.

Furthermore, the main-effects analysis revealed that surprisal extracted from GPT2, a large language model that was fine-tuned for Dutch using a context-window of 128 tokens (∼128 words), performed better in our TRF model of the data than surprisal calculated using a trigram model, despite both of the metrics performing well. This finding is in line with previous findings by Heilbron and colleagues (2019), who compared TRFs and reconstruction accuracy for trigram and GPT2 estimates in continuous listening in English. As in the present study, the authors showed that GPT2-derived surprisal estimates performed much better than trigram surprisal estimates.

An important open question is why GPT2-derived surprisal estimates perform better. Apparently, the probability the GPT2 model assigns to the next word is more related to brain activity in the delta band than the probability the trigram model assigns to that same word. Why is this the case? A possible reason for this finding is that transformers models such as GPT2 are better statistical approximators of the language input than simple models of (conditional) transitional probability, like a trigram model. One factor that contributes to better statistical approximation of the input and that is interpretable from a psychological point of view, is the amount of context that determines the state of the system—GPT2, the trigram model, and the brain—at the moment of processing an incoming word.

From a psychological perspective, a possibility is that the brain represents both long- and short-context distributional information during language comprehension (potentially independently from each other; Goodkind & Bicknell, 2021). The trigram model, with only two words of context, already captures variability in the neural signal, indicating that short-context distributional information is represented in the brain. Nevertheless, surprisal estimates from the fine-tuned GPT2 model are sensitive to variability at a distance of 128 tokens. This means that a word’s relation to the overall discourse is represented in those probability estimates, while this relation is hardly captured by probability at a short distance of two words. At the same time, GPT2 estimates do not exclude the probability of a word given the immediate context, as the two previous words are obviously part of the input to estimate surprisal for the current word. This means that GPT2 estimates of surprisal capture some of the same regularities as the trigram model. In that sense, GPT2 captures not only long-context effects, but also short-context effects. We can conclude that the results from the trigram and GPT2 models show that the delta-band neural signal covaries with surprisal estimates that find their origin in both short and long contexts during language comprehension.

### Computation of Structure in Time

The aim of this study was to assess whether the neural encoding of linguistic structure changes as a function of the distributional properties of a word, and whether this influence can be linked to probabilities in the immediate context (two preceding words) or rather to probabilities in the larger context (operationalized using GPT2). To this end, we extracted responses to annotations of syntactic structure, and we evaluated whether these responses differed between words that were statistically predictable (low surprisal) and words that were statistically relatively unpredictable (high surprisal).

The analysis revealed that distributional properties of a word affected the process of syntactic structure building in the temporal domain. Even after correcting for word duration, the response to a metric of syntactic structure—bottom-up node count—occurred earlier for words that were statistically predictable given the context (low surprisal) than for words that were unpredictable given the context (high surprisal). This effect was clearly visible using a simple, short-context metric of lexical distributional information: trigram surprisal. A cross-correlation on the grand average waveforms indicated that the neural signature of structure building occurred ∼150 ms earlier for low-surprisal words than for high-surprisal words. The temporal effect was slightly larger when using surprisal from GPT2, the operationalization of long-context surprisal; in this case, the neural signature of structure building was observed ∼190 ms earlier for low-surprisal words relative to high-surprisal words.

In an interactive, cascaded model of language comprehension, word recognition is hypothesized to occur prior to or simultaneously with the inference of syntactic structure (Marslen-Wilson & Welsh, 1978; Martin, 2016, 2020). Because words that are predictable from the context are recognized and read faster than words that are not predictable (Amenta et al., 2023; Aurnhammer & Frank, 2019; Grosjean & Itzler, 1984), it was deemed necessary to evaluate whether there is a difference in the time course of lexical processing between the high- and low-surprisal words. If such a difference existed in the data—or, more specifically, if signatures of lexical processing appeared earlier for low-surprisal than for high-surprisal words—it is possible that the effects observed for structure building do not reflect modulation of the structure building process by contextual distributional information directly. Instead, such a finding would open the possibility that contextual distributional information affects lexical processing in time, which could in turn affect structure building. However, a comparison between the high- and low-surprisal alternates of a response that has been related to lexical processing (word frequency; Slaats et al., 2023) revealed no temporal differences. In other words, the present analysis provided no evidence for temporal modulation of lexical processing as a consequence of contextual distributional information. This suggests that the temporal dynamics of lexical processing do not directly affect the process of structure building and makes it more likely that the contextual distributional information directly affects the process of structure building. However, that is not to say that lexical processing does not play a role: it is possible—and even likely—that other differences between processes at the lexical level that are not visible as delays will affect the process of structure building.

Taken together, these results indicate that the contextual probability of a word affects the computation of linguistic structure in time, with structural information being inferred either earlier or faster when a word is expected in a given statistical context. The last two words appear to be quite informative for this process, although longer context distributional information also plays a role.

### What and When Are Not Independent

The temporal effects shown in this study are in line with a model proposed by Ten Oever and Martin (2021, 2024). The model situates itself in the framework of neural oscillations serving a functional role in language comprehension. Besides activity in the delta and gamma bands outlined above, oscillatory activity in other frequency ranges has been suggested to play a key role in language processing, most notably the theta band (Doelling et al., 2014; Ghitza, 2013; Ghitza et al., 2013), but also the alpha and beta bands (Lam et al., 2016; Zioga et al., 2023), giving rise to various theories of the mechanisms underlying oscillations for language (e.g., Brennan & Martin, 2020; Meyer, 2018; Rimmele et al., 2018). An important open question in the formation of these theories is how ongoing oscillations can track language—a signal that is pseudorhythmic rather than purely rhythmic. Ten Oever and Martin (2021) propose that the pseudorhythmicity in speech carries information about the linguistic content. This works as follows. Imagine we are concerned with tracking the word rate. An ongoing oscillator tracks the average word rate. Now, the phase of the ongoing oscillation at which a word arrives, carries information about its predictability; if the word arrives early, that is, before the most excitatory moment in the cycle, the input is likely predictable from the context. On the other hand, if the input arrives relatively late—that is, after the most excitatory moment in the cycle—the word is likely to be less predictable from the context. This allows the language system of the comprehender to anticipate unpredictable input.

Obviously, the current study does not speak to this directly, as our readout does not provide information about phase, and our study concerns high-level linguistic operations, which are not (yet) explicitly embedded in the model the authors proposed. What the present results do indicate is that contextual information not only affects the timing of word production but also affects the timing of higher level operations. This is much in line with what Ten Oever & Martin suggest; an extension of their proposal, perhaps. Ten Oever and Martin (2021) suggest that a neural population that corresponds to a linguistic unit in the internal language model of an individual (their individually acquired structural and statistical knowledge of language) may be sensitized if that exact linguistic unit is predictable from the context. By consequence, this population may be active earlier, on a less excitable phase of the ongoing oscillation. According to the present results, lexical distributional information does not necessarily activate neural populations that represent lexical information earlier (relative to word onset). A higher lexical probability does, however, more quickly activate neural populations that play a role in representing the syntactic structure underlying the input.

### Long- and Short-Context Effects on Structure Building

Interestingly, most of the temporal delay or shift that we observe in the high and low surprisal node count responses is captured by the simple trigram models (150 ms). This suggests that local statistical relations between words have a large impact on the process of syntactic structure building. However, not all of the temporal effect is captured by simple trigram surprisal estimates; GPT2-based models suggest that the temporal difference in the response to bottom-up node count can be as large as 190 ms. Why does this difference exist? We propose that short-context statistical relations are the strongest cue for structure building. Nevertheless, a statistical context of two words as in the case of the trigram model is minimal. Statistical patterns associated with dependencies within a sentence, such as subject–verb relations with intervening material, are not captured. At the same time, the short-context statistical relations may be affected by probability in the discourse context. The fine-tuning of GPT2 for Dutch used a context of 128 tokens, which means that surprisal estimates are sensitive to words that appeared less than 128 words ago. This means that the surprisal estimates from GPT2 are sensitive to some within-sentence structural dependencies that the trigram model fails to capture. In addition, our stimuli were fairytales. This means that they contained words and word sequences that are locally unpredictable, but globally predictable. For example, in one of the stories, the main character is a duckling that can speak (“[…], zei het eendje,” which translates to “[…], said the duckling”). We situate these findings with those from Nieuwland and Van Berkum (2006), who show that the discourse context can eliminate N400 effects in sentences with anomalies of animacy (e.g., “the peanut was in love”). Importantly, the fact that GPT2 captures regularity in the global context and humans do, too (and trigram models do not), does not mean that the mechanism underlying this representation is shared or even necessarily similar between GPT2 and humans (Guest & Martin, 2023).

### Word Duration, Surprisal Estimate, and Response Amplitude

Besides the clear finding of a temporal delay as a function of surprisal that does not depend on word duration, and is not a direct consequence of temporal delays at the lexical level as a function of surprisal, we observe a pattern in the response amplitude and explained variance that appears to depend on word duration. The pattern is as follows: Before correction for word duration, we observed a larger amplitude and larger variance explained for high surprisal than for low surprisal in the trigram models. These differences did not (clearly) exist in the GPT2 models. After correction for word duration, the pattern shifts: there are no clear differences between high and low surprisal response amplitude and variance explained for the trigram models. In the GPT2 models, however, we find larger response ampitude for low surprisal than for high surprisal bottom-up node count responses and a similar effect on the variance explained. If we group these findings for simplicity, we can conclude that correcting for word duration decreases the amplitude for the high-surprisal words. The parallel between response amplitude and variance explained suggests that they are connected; it is possible that a response explains more of the variance in the signal, if it has a larger amplitude.

These findings confirm that there is a relationship between surprisal and word duration, which has been known for a while (Mahowald et al., 2013; Piantadosi et al., 2011). Beyond this, however, it also suggests that word duration and surprisal together drive response amplitudes to higher level features such as syntactic structure building. The present data do not allow us to draw conclusions about this relation, though there are several possibilities for how the factors relate to each other. It is important to keep in mind that lexical surprisal contains influences from different latent factors, one being syntactic structure (Slaats & Martin, 2023). Syntactic predictability has been found to affect the duration of utterances, with less predictable structures yielding longer utterances (Kuperman & Bresnan, 2012; Moore-Cantwell, 2013). It is possible, then, that the duration of the word is itself a cue toward the syntactic structure, and by proxy, it is possible that we have affected the syntactic predictability of the words and constituents in the high and low surprisal categories. What effect this variable itself should have on the neural response to bottom-up node counts is unclear, although our results suggest that the effect is mostly one of response amplitude, with less predictable, longer words receiving larger amplitudes. Studies with highly controlled stimuli may provide further insight into these relationships. Here, we wish to suggest only that many different aspects of the stimulus, even its duration, likely play a role in high-level stages of the process of language comprehension (see also Martin, 2016).

### Conclusion

Over the past several decades, much psycholinguistic research has focused on accounting for syntactic phenomena as a form of transitional probabilities between different linguistic units (e.g., Frank & Bod, 2011; Frank & Christiansen, 2018; Frost et al., 2019; McCauley & Christiansen, 2019), or as a level of representation that is hierarchically structured and abstracts away from the lexical items (e.g., Brennan & Hale, 2019; Lo et al., 2022; Matchin & Hickok, 2020). While it is clear that both types of linguistic knowledge must play a role (Maheu et al., 2022; Roark et al., 2009), the mechanistic relationship between these representations remains unclear. In this study, we aimed to test a framework where humans use lexical distributional information to build abstract, hierarchical representations that give rise to meaning as an instance of cue integration. Specifically, we asked whether the low-frequency neural encoding of linguistic structure changes as a function of the distributional properties of a word, and whether this influence can be linked to probabilities in the immediate context (two preceding words) or rather to probabilities in the larger context (operationalized using GPT2). We did this by extracting delta-band responses to syntactic node count using TRFs, and comparing these responses between high- and low surprisal words. Our results showed that lexical distributional information indeed affects the process of syntactic structure building as indexed by delta-band neural responses to node count, and that it did so in the temporal domain: the delta-band response to structure building was delayed by 150 to 190 ms for words that are statistically unpredictable given the context (high surprisal) relative to words that are statistically predictable given the context. This delay appeared not to be driven by temporal changes in lexical processing as indexed by word frequency. In addition, we have shown that most of this effect is captured when using trigram surprisal (150 out of a maximum 190 ms). Our findings speak to theories that model language comprehension as a cascaded process in which cues at different levels are used to infer higher level representations (Marslen-Wilson & Welsh, 1978; Martin, 2016, 2020), and theories that link abstract linguistic knowledge to the temporal properties of speech (Ten Oever & Martin, 2021, 2024).

## ACKNOWLEDGMENTS

We thank Hugo Weissbart for advice on the use of temporal response functions; Cas Coopmans and Michelle Suijkerbuijk for help creating the syntactic annotations; Ryan Law, Ioanna Zioga, Hugo Weissbart, Filiz Tezcan Semerci, Lorenzo Titone, and Cas Coopmans for contributing to data acquisition; and the members of the Language and Computation in Neural Systems (LaCNS) research group and the Psychology of Language department at the Max Planck Institute for Psycholinguistics for valuable input on earlier versions of this project.

## FUNDING INFORMATION

Andrea E. Martin, Nederlandse Organisatie voor Wetenschappelijk Onderzoek (https://dx.doi.org/10.13039/501100003246), Award ID: 016.Vidi.188.029. Andrea E. Martin, Nederlandse Organisatie voor Wetenschappelijk Onderzoek (https://dx.doi.org/10.13039/501100003246), Award ID: 024.001.006. Andrea E. Martin, Independent Max Planck Research Group. Andrea E. Martin, Lise Meitner Research Group Language and Computation in Neural Systems.

## AUTHOR CONTRIBUTIONS

**Sophie Slaats**: Conceptualization; Formal analysis; Methodology; Project administration; Visualization; Writing – original draft; Writing – review & editing. **Antje S. Meyer**: Conceptualization; Methodology; Supervision; Writing – review & editing. **Andrea E. Martin**: Conceptualization; Methodology; Supervision; Writing – review & editing.

## Supplementary Material


