# Supplementary material for: Lexical Surprisal Shapes the Time Course of Syntactic Structure Building
Source: Neurobiol Lang (Camb). 2024 Oct 11;5(4):942–80. doi: 10.1162/nol_a_00155 (PMC11556436; doi:10.1162/nol_a_00155)
Supplement: Supplementary file 1 [file nol-5-4-942-s001.pdf]

Appendix A. Correlation matrices for feature values

1. Trigram models

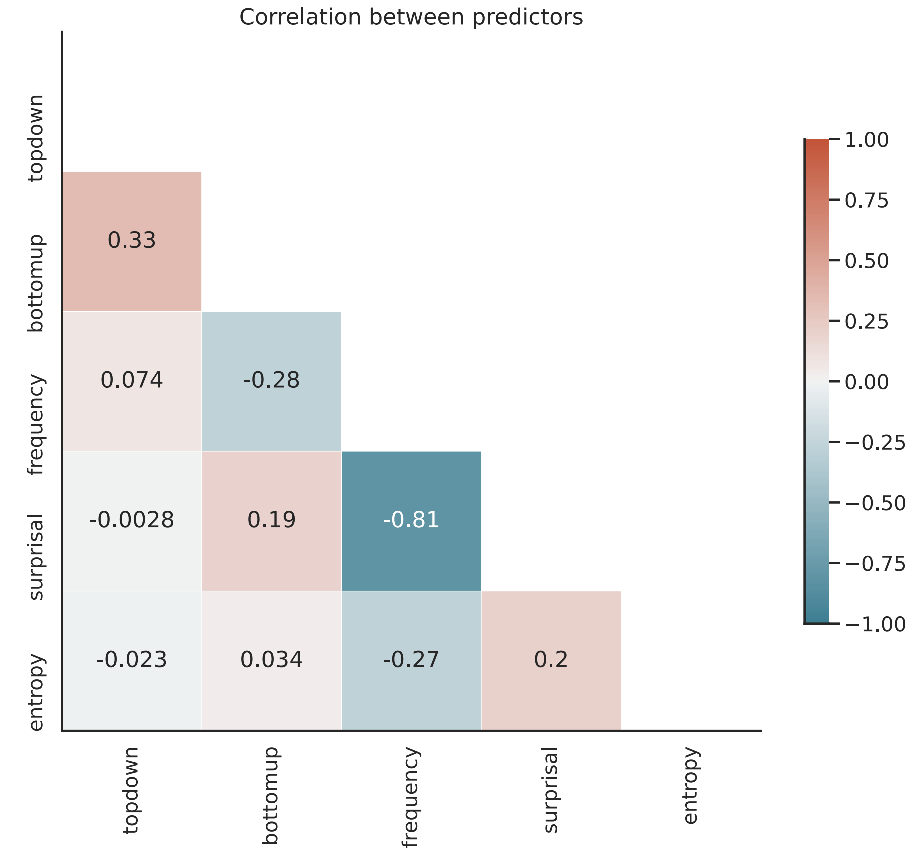

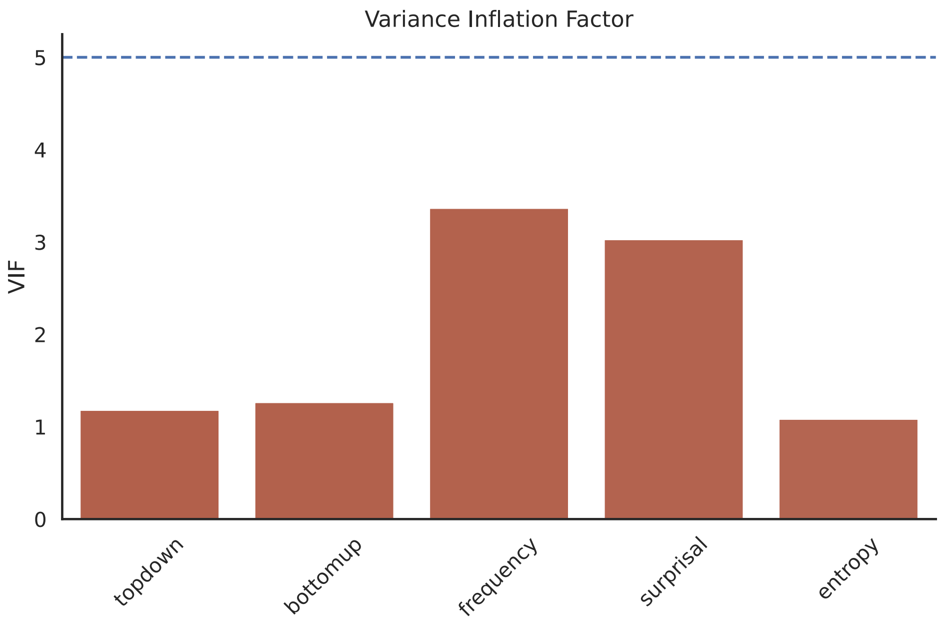

2. GPT2-models

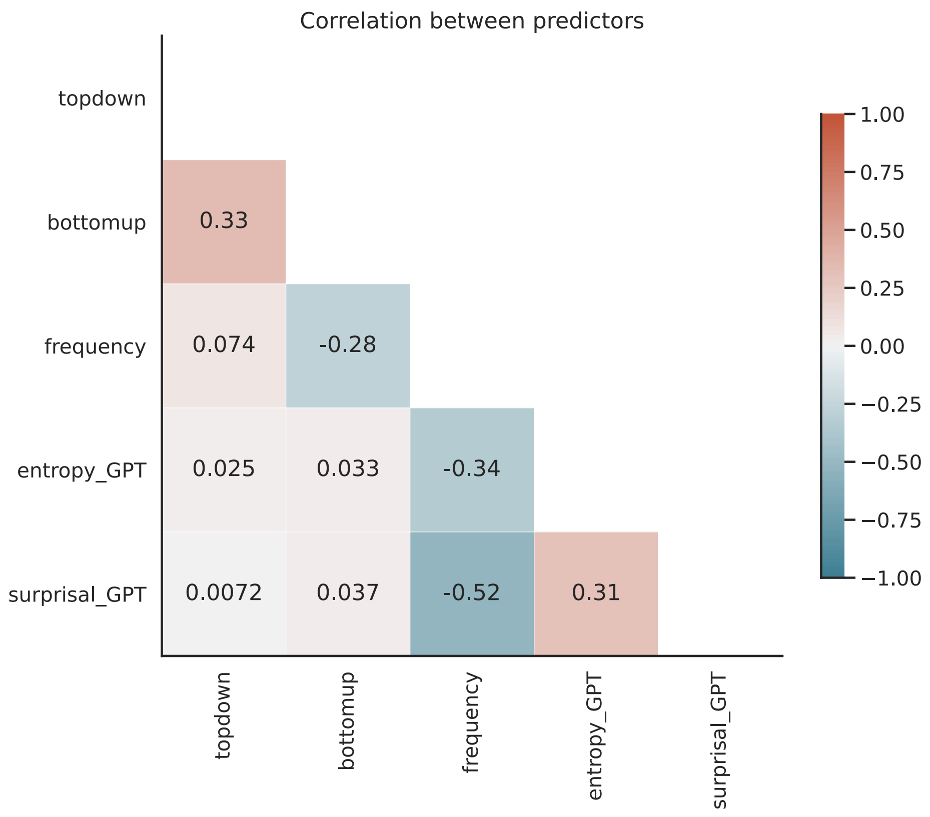

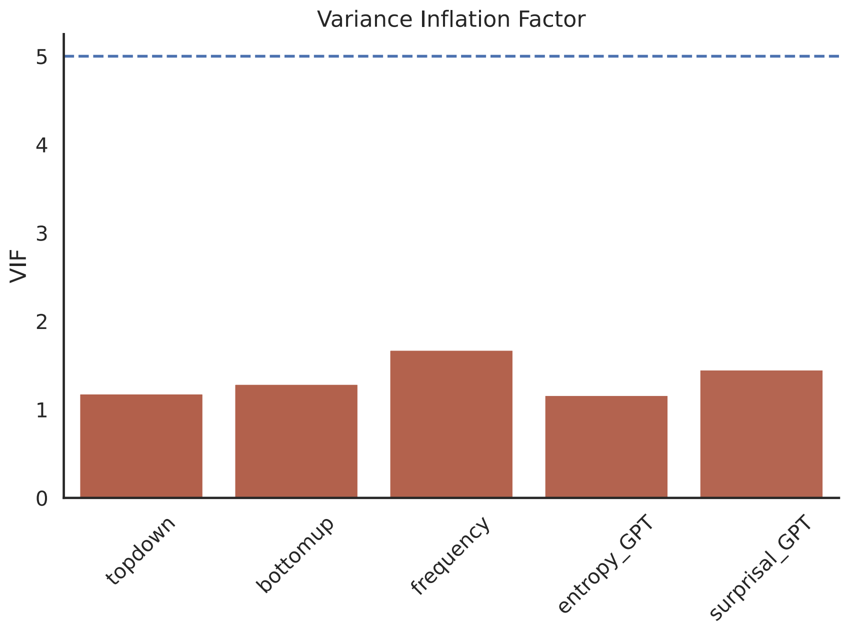

Appendix B. Model comparison statistics as output by *step* (LmerTest) from the 'Main effects' analysis

1. Trigram models

1.1. Random effects structure: 1 + top down * bottom up * surprisal | participant

1.1.1. Random effects

Effect	Eliminated params	npar	logLik	AIC	LRT	df	p value (χ^2)
<none>		53	2177.04	-4248.07			
topdown * bottomup * surprisal in (1 + topdown * bottomup * surprisal | subject)	0	45	2163.49	-4236.99	27.08	8	$6.84e^{-04}$

1.1.2. Fixed effects

Effect	Elimin. params	Sum.Sq	NumDF	DenDF	F value	p value
entropy surprisal * topdown * bottomup	1	$7.52e^{-08}$	1	226.28	0.80	0.37
entropy * surprisal * bottomup	2	$1.00e^{-08}$	1	253.98	0.11	0.74
entropy * surprisal * topdown	3	$1.33e^{-08}$	1	232.02	0.14	0.71
surprisal * topdown * bottomup	4	$1.44e^{-08}$	1	40.41	0.15	0.70
entropy * topdown * bottomup	5	$2.43e^{-08}$	1	233.10	0.26	0.61
surprisal * bottomup	6	$6.15e^{-08}$	1	50.27	0.67	0.42
entropy * bottomup	7	$1.20e^{-07}$	1	235.96	1.30	0.26
entropy * surprisal	0	$6.06e^{-06}$	1	236.97	65.60	$2.92e^{-14}$
entropy * topdown	0	$5.33e^{-07}$	1	236.97	5.77	0.02
surprisal * topdown	0	$1.59e^{-06}$	1	24.72	17.19	$3.47e^{-04}$
topdown * bottomup	0	$9.03e^{-06}$	1	43.25	97.85	$1.12e^{-12}$

1.2. Random effects structure: 1 + top down * bottom up * entropy | participant

1.2.1. Random effects

Effect	Eliminated params	npar	logLik	AIC	LRT	df	p value (χ^2)
<none>		53	2097.24	-4088.48			
topdown * bottomup * entropy in (1 + topdown * bottomup * entropy | subject)	1	45	2097.08	-4104.16	0.32	8	1.00
topdown * entropy in (topdown + bottomup + entropy + topdown * bottomup + topdown * entropy + bottomup * entropy | subject)	2	38	20967.00	-4118.00	0.16	7	1.00
bottomup * entropy in (topdown + bottomup + entropy + topdown * bottomup + bottomup * entropy | subject)	3	32	2096.57	-4129.14	0.85	6	0.99
topdown * bottomup in (topdown + bottomup + entropy + topdown * bottomup | subject)	4	27	2094.84	-4135.68	3.47	5	0.63
topdown in (topdown + bottomup + entropy | subject)	0	23	2050.64	-4055.27	88.41	4	$2.87e^{-18}$
bottomup in (topdown + bottomup + entropy | subject)	0	23	1893.74	-3741.49	402.19	4	$9.35e^{-86}$
entropy in (topdown + bottomup + entropy | subject)	0	23	2088.14	-4130.27	13.40	4	$9.46e^{-03}$

1.2.2. Fixed effects

Effect	Elimin. params	Sum.Sq	NumDF	DenDF	F value	p value
entropy * surprisal * topdown * bottomup	1	$7.52e^{-08}$	1	276.00	0.38	0.54
entropy * surprisal * bottomup	2	$1.00e^{-08}$	1	277.00	0.05	0.82
entropy * surprisal * topdown	3	$1.33e^{-08}$	1	298.59	0.05	0.82
surprisal * topdown * bottomup	4	$2.03e^{-08}$	1	279.00	0.10	0.75
entropy * topdown * bottomup	5	$2.43e^{-08}$	1	298.53	0.09	0.76
surprisal * bottomup	6	$6.15e^{-08}$	1	281.00	0.32	0.57
entropy * bottomup	7	$1.20e^{-07}$	1	282.00	0.62	0.43
entropy * topdown	8	$5.33e^{-07}$	1	283.00	2.77	0.10
entropy * surprisal	0	$6.06e^{-06}$	1	284.00	31.31	$5.19e^{-08}$
surprisal * topdown	0	$2.78e^{-06}$	1	284.00	14.36	$1.84e^{-04}$
topdown * bottomup	0	$1.42e^{-05}$	1	284.00	73.22	$7.39e^{-16}$

1.3. Random effects structure: 1 + top down * surprisal * entropy | participant

1.3.1. Random effects

Effect	Eliminated params	npar	logLik	AIC	LRT	df	p value (χ^2)
<none>		53	1898.36	-3690.72			
topdown * surprisal * entropy in (1 + topdown * surprisal * entropy | subject)	1	45	1898.31	-3706.63	0.09	8	1
surprisal * entropy in (topdown + surprisal + entropy + topdown * surprisal + topdown * entropy + surprisal * entropy | subject)	2	38	1898.29	-3720.59	0.04	7	1
topdown * entropy in (topdown + surprisal + entropy + topdown * surprisal + topdown * entropy | subject)	3	32	1898.28	-3732.56	0.02	6	1
topdown * surprisal in (topdown + surprisal + entropy + topdown * surprisal | subject)	4	27	1898.17	-3742.34	0.22	5	1
entropy in (topdown + surprisal + entropy | subject)	5	23	1897.68	-3749.35	0.99	4	0.91
topdown in (topdown + surprisal | subject)	0	20	1889.76	-3739.52	15.83	3	$1.23e^{-03}$
surprisal in (topdown + surprisal | subject)	0	20	1893.43	-3746.85	8.50	3	0.04

1.3.2. Fixed effects

Effect	Elimin. params	Sum.Sq	NumDF	DenDF	F value	p value
entropy * surprisal * topdown * bottomup	1	$7.52e^{-08}$	1	322.00	0.08	0.77
entropy * surprisal * bottomup	2	$1.00e^{-08}$	1	263.16	0.01	0.92
entropy * surprisal * topdown	3	$1.33e^{-08}$	1	324.00	0.01	0.90
surprisal * topdown * bottomup	4	$2.03e^{-08}$	1	325.00	0.02	0.88
entropy * topdown * bottomup	5	$2.43e^{-08}$	1	326.00	0.03	0.87
surprisal * bottomup	6	$6.15e^{-08}$	1	327.00	0.07	0.79
entropy * bottomup	7	$1.20e^{-07}$	1	328.00	0.14	0.71
entropy * topdown	8	$5.33e^{-07}$	1	329.00	0.60	0.44
surprisal * topdown	9	$2.78e^{-06}$	1	330.00	3.14	0.08
entropy * surprisal	0	$6.06e^{-06}$	1	287.85	6.53	0.01
topdown * bottomup	0	$1.42e^{-05}$	1	287.85	15.27	$1.16e^{-04}$

1.4. Random effects structure: 1 + bottom up * surprisal * entropy | participant

1.4.1. Random effects

Effect	Eliminated params	npar	logLik	AIC	LRT	df	p value (χ^2)
<none>		53	2091.65	-4077.30			
bottomup * surprisal * entropy in (1 + bottomup * surprisal * entropy | subject)	0	45	1967.07	-3844.15	249.15	8	$2.61e^{-49}$

1.4.2. Fixed effects

Effect	Elimin. params	Sum.Sq	NumDF	DenDF	F value	p value
entropy * surprisal * topdown * bottomup	1	$7.52e^{-08}$	1	275.92	0.38	0.54
entropy * surprisal * bottomup	2	$9.90e^{-09}$	1	224.52	0.05	0.82
entropy * surprisal * topdown	3	$1.33e^{-08}$	1	277.97	0.07	0.79
surprisal * topdown * bottomup	4	$2.03e^{-08}$	1	278.98	0.10	0.75
entropy * topdown * bottomup	5	$2.43e^{-08}$	1	279.99	0.13	0.72
surprisal * bottomup	6	$5.78e^{-08}$	1	110.22	0.30	0.59
entropy * bottomup	7	$1.13e^{-07}$	1	157.34	0.59	0.44
entropy * topdown	8	$5.33e^{-07}$	1	282.84	2.77	0.10
entropy * surprisal	0	$5.04e^{-06}$	1	62.02	26.06	$3.39e^{-06}$
surprisal * topdown	0	$2.78e^{-06}$	1	283.81	14.37	$1.83e^{-04}$
topdown * bottomup	0	$1.42e^{-05}$	1	283.81	73.27	$7.24e^{-16}$

1.5. Best models for every random effects structure configuration & their AIC-value

Largest model	Chosen model	df	AIC
r_values ~ entropy * surprisal * topdown * bottomup + (1 + topdown * bottomup * surprisal | subject)	r_values ~ entropy + surprisal + topdown + bottomup + entropy * surprisal + surprisal * topdown + topdown * bottomup + (1 + topdown * bottomup * surprisal | subject)	46	-4372.50
r_values ~ entropy * surprisal * topdown * bottomup + (1 + topdown * bottomup * entropy | subject)	r_values ~ entropy + surprisal + topdown + bottomup + entropy * surprisal + surprisal * topdown + topdown * bottomup + (topdown + bottomup + entropy | subject)	19	-4273.29
r_values ~ entropy * surprisal * topdown * bottomup + (1 + topdown * surprisal * entropy | subject)	r_values ~ entropy * surprisal + topdown * bottomup + (topdown + surprisal | subject)	14	-3885.84
r_values ~ entropy * surprisal * topdown * bottomup + (1 + bottomup * surprisal * entropy | subject)	r_values ~ entropy * surprisal + topdown * bottomup + surprisal * topdown (1 + bottomup * surprisal * entropy | subject)	45	-4214.86

2. GPT2 models

2.1. Random effects structure: 1 + top down * bottom up * surprisal | participant

2.1.1. Random effects

Effect	Eliminated params	npar	logLik	AIC	LRT	df	p value (χ^2)
<none>		53	2122.21	-4138.41			
topdown * bottomup * surprisal in (1 + topdown * bottomup * surprisal | subject)	1	45	2121.76	-4153.52	0.89	8	1.00
topdown * bottomup in (topdown + bottomup + surprisal + topdown * bottomup + topdown * surprisal + bottomup * surprisal | subject)	2	38	2120.75	-4165.49	2.03	7	0.96
topdown * surprisal in (topdown + bottomup + surprisal + topdown * surprisal + bottomup * surprisal | subject)	3	32	2118.98	-4173.96	3.53	6	0.74
topdown in (topdown + bottomup + surprisal + bottomup * surprisal | subject)	0	27	2062.52	-4071.05	112.91	5	$9.94e^{-23}$
bottomup * surprisal in (topdown + bottomup + surprisal + bottomup * surprisal | subject)	0	27	2113.00	-4172.00	11.96	5	0.04

2.1.2. Fixed effects

Effect	Elimin. params	Sum.Sq	NumDF	DenDF	F value	p value
entropy * surprisal * topdown * bottomup	1	$5.46e^{-08}$	1	276.00	0.39	0.53
entropy * surprisal * topdown	2	$3.29e^{-09}$	1	295.37	0.01	0.90
surprisal * topdown * bottomup	3	$5.97e^{-09}$	1	300.20	0.03	0.87
entropy * topdown * bottomup	4	$5.48e^{-08}$	1	278.99	0.39	0.53
entropy * surprisal * bottomup	5	$4.59e^{-07}$	1	302.44	2.08	0.15
surprisal * bottomup	6	$1.03e^{-08}$	1	37.23	0.07	0.79
entropy * bottomup	7	$2.98e^{-07}$	1	281.83	2.13	0.15
entropy * surprisal	0	$1.63e^{-06}$	1	282.83	11.60	$7.54e^{-04}$
entropy * topdown	0	$9.27e^{-07}$	1	282.83	6.59	0.01
surprisal * topdown	0	$5.58e^{-07}$	1	282.83	3.97	0.05
topdown * bottomup	0	$1.71e^{-05}$	1	282.83	121.30	$1.04e^{-23}$

2.2. Random effects structure: 1 + top down * bottom up * entropy | participant

2.2.1. Random effects

Effect	Eliminated params	npar	logLik	AIC	LRT	df	p value (χ^2)
<none>		53	1999.12	-3892.23			
topdown * bottomup * entropy in (1 + topdown * bottomup * entropy | subject)	0	45	1990.44	-3890.87	17.36	8	0.03

2.2.2. Fixed effects

Effect	Elimin. params	Sum.Sq	NumDF	DenDF	F value	p value
entropy * surprisal * topdown * bottomup	1	$5.46e^{-08}$	1	275.97	0.15	0.70
entropy * surprisal * topdown	2	$3.29e^{-09}$	1	272.02	$8.28e^{-02}$	0.93
surprisal * topdown * bottomup	3	$5.97e^{-09}$	1	277.99	0.02	0.90
entropy * topdown * bottomup	4	$5.18e^{-08}$	1	110.16	0.14	0.71
entropy * surprisal * bottomup	5	$4.59e^{-07}$	1	279.02	1.23	0.27
surprisal * bottomup	6	$1.59e^{-08}$	1	280.96	0.04	0.84
entropy * bottomup	7	$2.42e^{-07}$	1	98.84	0.62	0.43
surprisal * topdown	8	$5.58e^{-07}$	1	282.71	1.51	0.22
entropy * topdown	9	$9.31e^{-07}$	1	127.57	2.37	0.13
entropy * surprisal	0	$1.63e^{-06}$	1	283.96	4.40	0.04
topdown * bottomup	0	$1.63e^{-05}$	1	171.87	44.00	$4.10e^{-10}$

2.3. Random effects structure: 1 + top down * surprisal * entropy | participant

2.3.1.Random effects

Effect	Eliminated params	npar	logLik	AIC	LRT	df	p value (χ^2)
<none>		53	1899.59	-3693.18			
topdown * surprisal * entropy in (1 + topdown * surprisal * entropy | subject)	1	45	1899.48	-3708.96	0.23	8	1.00
surprisal * entropy in (topdown + surprisal + entropy + topdown * surprisal + topdown * entropy + surprisal * entropy | subject)	2	38	1899.47	-3722.94	0.02	7	1.00
topdown * entropy in (topdown + surprisal + entropy + topdown * surprisal + topdown * entropy | subject)	3	32	1899.41	-3734.83	0.11	6	1.00
topdown * surprisal in (topdown + surprisal + entropy + topdown * surprisal | subject)	4	27	1899.15	-3744.3	0.53	5	0.99
entropy in (topdown + surprisal + entropy | subject)	5	23	1897.15	-3748.29	4.00	4	0.41
topdown in (topdown + surprisal | subject)	6	20	1894.13	-3748.27	6.02	3	0.11
surprisal in (surprisal | subject)	0	18	1876.60	-3717.20	35.07	2	$2.42e^{-08}$

2.3.2.Fixed effects

Effect	Elimin. params	Sum.Sq	NumDF	DenDF	F value	p value
entropy * surprisal * topdown * bottomup	1	$5.46e^{-08}$	1	322.00	0.06	0.80
entropy * surprisal * topdown	2	$3.29e^{-09}$	1	323.00	$3.72e^{-02}$	0.95
surprisal * topdown * bottomup	3	$5.97e^{-09}$	1	324.00	$6.75e^{-02}$	0.93
entropy * topdown * bottomup	4	$5.48e^{-08}$	1	325.00	0.06	0.80
entropy * surprisal * bottomup	5	$4.59e^{-07}$	1	326.00	0.52	0.47
surprisal * bottomup	6	$1.59e^{-08}$	1	327.00	0.02	0.89
entropy * bottomup	7	$2.98e^{-07}$	1	328.00	0.34	0.56
surprisal * topdown	8	$5.58e^{-07}$	1	329.00	0.64	0.42
entropy * topdown	9	$9.27e^{-07}$	1	330.00	1.06	0.30
entropy * surprisal	10	$1.63e^{-06}$	1	331.00	1.87	0.17
entropy	11	$2.47e^{-06}$	1	332.00	2.83	0.09
surprisal	0	$4.65e^{-05}$	1	23.00	52.93	$2,1e^{-07}$
topdown * bottomup	0	$1.71e^{-05}$	1	333.00	19.41	$1,43e^{-05}$

2.4. Random effects structure: 1 + bottom up * surprisal * entropy | participant

2.4.1. Random effects

Effect	Eliminated params	npar	logLik	AIC	LRT	df	p value (χ^2)
<none>		53	2085.14	-4064.29			
bottomup * surprisal * entropy in (1 + bottomup * surprisal * entropy | subject)	1	45	2084.99	-4079.98	0.30	8	1.00
surprisal * entropy in (bottomup + surprisal + entropy + bottomup * surprisal + bottomup * entropy + surprisal * entropy | subject)	2	38	2083.37	-4090.75	3.24	7	0.86
bottomup * entropy in (bottomup + surprisal + entropy + bottomup * surprisal + bottomup * entropy | subject)	3	32	2081.03	-4098.06	4.68	6	0.58
bottomup * surprisal in (bottomup + surprisal + entropy + bottomup * surprisal | subject)	4	27	2077.69	-4101.38	6.68	5	0.25
bottomup in (bottomup + surprisal + entropy | subject)	0	23	1895.11	-3744.23	365.15	4	$9.38e^{-78}$
surprisal in (bottomup + surprisal + entropy | subject)	0	23	1980.33	-3914.66	194.72	4	$5.12e^{-41}$
entropy in (bottomup + surprisal + entropy | subject)	0	23	2059.91	-4073.82	35.56	4	$3.57e^{-07}$

2.4.2. Fixed effects

Effect	Elimin. params	Sum.Sq	NumDF	DenDF	F value	p value
entropy * surprisal * topdown * bottomup	1	$5.46e^{-08}$	1	276.00	0.28	0.60
entropy * surprisal * topdown	2	$3.29e^{-09}$	1	277.00	0.02	0.90
surprisal * topdown * bottomup	3	$5.97e^{-09}$	1	278.00	0.03	0.86
entropy * topdown * bottomup	4	$5.48e^{-08}$	1	279.00	0.28	0.60
entropy * surprisal * bottomup	5	$4.59e^{-07}$	1	280.00	2.36	0.13
surprisal * bottomup	6	$1.59e^{-08}$	1	281.00	0.08	0.78
entropy * bottomup	7	$2.98e^{-07}$	1	282.00	1.53	0.22
surprisal * topdown	8	$5.58e^{-07}$	1	283.00	2.86	0.09
entropy * surprisal	0	$1.63e^{-06}$	1	284.00	8.29	$4.28e^{-03}$
entropy * topdown	0	$9.27e^{-07}$	1	284.00	4.71	0.03
topdown * bottomup	0	$1.71e^{-05}$	1	284.00	86.70	$3.6e^{-18}$

2.5. Best models for every random effects structure configuration & their AIC-value

Largest model	Chosen model	df	AIC
r_values ~ entropy * surprisal * topdown * bottomup + (1 + topdown * bottomup * surprisal | subject)	r_values ~ entropy + surprisal + topdown + bottomup + entropy * surprisal + entropy * topdown + surprisal * topdown + topdown * bottomup + (topdown + bottomup * surprisal | subject)	25	-4292.74
r_values ~ entropy * surprisal * topdown * bottomup + (1 + topdown * bottomup * entropy | subject)	r_values ~ entropy + surprisal + topdown + bottomup + entropy * surprisal + topdown * bottomup (1 + topdown * bottomup * entropy | subject)	44	-4041.09
r_values ~ entropy * surprisal * topdown * bottomup + (1 + topdown * surprisal * entropy | subject)	r_values ~ surprisal + topdown * bottomup + (surprisal | subject)	9	-3924.08
r_values ~ entropy * surprisal * topdown * bottomup + (1 + bottomup * surprisal * entropy | subject)	r_values ~ entropy + surprisal + topdown + bottomup + entropy * surprisal + entropy * topdown + topdown * bottomup + (bottomup + surprisal + entropy | subject)	19	-4235.94

3. Trigram vs GPT2

In the tables below, the variable 'lm' stands for 'language model'.

3.1. Random effects structure: 1 + entropy * surprisal | participant

3.1.1. Random effects

Effect	Eliminated params	npar	logLik	AIC	LRT	df	p value (χ^2)
<none>		19	985.01	-1932.02			
entropy * surprisal in (1 + entropy * surprisal | subject)	1	15	984.90	-1939.80	0.22	4	0.99
entropy in (entropy + surprisal | subject)	2	12	984.31	-1944.61	1.19	3	0.76
surprisal in (surprisal | subject)	0	10	972.00	-1924.01	24.61	2	$4.54e^{-06}$

3.1.2. Fixed effects

Effect	Elimin. params	Sum.Sq	NumDF	DenDF	F value	p value
lm * entropy * surprisal	1	$5.49e^{-07}$	1	138.00	1.84	0.18
lm * entropy	2	$8.78e^{-07}$	1	139.00	2.93	0.09
entropy * surprisal	3	$1.08e^{-06}$	1	140.00	3.56	0.06
entropy	0	$4.74e^{-06}$	1	141.00	15.34	$1.40e^{-04}$
lm * surprisal	0	$6.69e^{-06}$	1	141.00	21.63	$7.55e^{-06}$

3.2. Random effects structure: 1 + model * entropy | participant

3.2.1. Random effects

Effect	Eliminated params	npar	logLik	AIC	LRT	df	p value (χ^2)
<none>		19	977.36	-1916.71			
lm * entropy in (1 + lm * entropy | subject)	1	15	975.83	-1921.66	3.06	4	0.55
entropy in (lm + entropy | subject)	2	12	974.77	-1925.55	2.11	3	0.55
lm in (lm | subject)	0	10	972.00	-1924.01	5.54	2	0.06

3.2.2. Fixed effects

Effect	Elimin. params	Sum.Sq	NumDF	DenDF	F value	p value
lm * entropy * surprisal	1	$5.49e^{-07}$	1	138.00	1.52	0.22
lm * entropy	2	$8.78e^{-07}$	1	139.00	2.42	0.12
entropy * surprisal	3	$1.08e^{-06}$	1	140.00	2.96	0.09
entropy	0	$4.74e^{-06}$	1	141.00	12.77	$4.82e^{-04}$
lm * surprisal	0	$6.69e^{-06}$	1	141.00	18.01	$3.96e^{-05}$

3.3. Random effects structure: 1 + model * surprisal | participant

3.3.1. Random effects

Effect	Eliminated params	npar	logLik	AIC	LRT	df	p value (χ^2)
<none>		19	1023.36	-2008.73			
lm * surprisal in (1 + lm * surprisal | subject)	0	15	999.73	-1969.47	47.26	4	$1.35e^{-09}$

3.3.2. Fixed effects

Effect	Elimin. params	Sum.Sq	NumDF	DenDF	F value	p value
lm * entropy * surprisal	0	$5.49e^{-07}$	1	115.00	4.27	0.04

3.4. Best models for every random effects structure configuration and their AIC-value

Largest model	Chosen model	df	AIC
r_values ~ lm * entropy * surprisal + (1 + entropy * surprisal | subject)	r_values ~ lm * surprisal + entropy + (surprisal | subject)	9	-1987.92
r_values ~ lm * entropy * surprisal + (1 + lm * entropy | subject)	r_values ~ lm * surprisal + entropy + surprisal + (lm | subject)	9	-1969.71
r_values ~ lm * entropy * surprisal + (1 + lm * surprisal | subject)	r_values ~ lm * entropy * surprisal + (1 + lm * surprisal | subject)	19	-2007.73
